# The genomic and epigenetic footprint of local adaptation to variable climates in kiwifruit

**DOI:** 10.1093/hr/uhad031

**Published:** 2023-02-21

**Authors:** Xu Zhang, Rui Guo, Ruinan Shen, Jacob B Landis, Quan Jiang, Fang Liu, Hengchang Wang, Xiaohong Yao

**Affiliations:** CAS Key Laboratory of Plant Germplasm Enhancement and Specialty Agriculture, Wuhan Botanical Garden, the Chinese Academy of Sciences, Wuhan 430074, Hubei, China; College of Life Sciences, University of Chinese Academy of Sciences, Beijing 100049, China; CAS Key Laboratory of Plant Germplasm Enhancement and Specialty Agriculture, Wuhan Botanical Garden, the Chinese Academy of Sciences, Wuhan 430074, Hubei, China; College of Life Sciences, University of Chinese Academy of Sciences, Beijing 100049, China; CAS Key Laboratory of Plant Germplasm Enhancement and Specialty Agriculture, Wuhan Botanical Garden, the Chinese Academy of Sciences, Wuhan 430074, Hubei, China; College of Life Sciences, University of Chinese Academy of Sciences, Beijing 100049, China; School of Integrative Plant Science, Section of Plant Biology and the L.H. Bailey Hortorium, Cornell University, Ithaca, NY 14853 USA; BTI Computational Biology Center, Boyce Thompson Institute, Ithaca, NY 14853, USA; CAS Key Laboratory of Plant Germplasm Enhancement and Specialty Agriculture, Wuhan Botanical Garden, the Chinese Academy of Sciences, Wuhan 430074, Hubei, China; College of Life Sciences, University of Chinese Academy of Sciences, Beijing 100049, China; CAS Key Laboratory of Plant Germplasm Enhancement and Specialty Agriculture, Wuhan Botanical Garden, the Chinese Academy of Sciences, Wuhan 430074, Hubei, China; CAS Key Laboratory of Plant Germplasm Enhancement and Specialty Agriculture, Wuhan Botanical Garden, the Chinese Academy of Sciences, Wuhan 430074, Hubei, China; CAS Key Laboratory of Plant Germplasm Enhancement and Specialty Agriculture, Wuhan Botanical Garden, the Chinese Academy of Sciences, Wuhan 430074, Hubei, China

## Abstract

A full understanding of adaptive genetic variation at the genomic level will help address questions of how organisms adapt to diverse climates. *Actinidia eriantha* is a shade-tolerant species, widely distributed in the southern tropical region of China, occurring in spatially heterogeneous environments. In the present study we combined population genomic, epigenomic, and environmental association analyses to infer population genetic structure and positive selection across a climatic gradient, and to assess genomic offset to climatic change for *A. eriantha*. The population structure is strongly shaped by geography and influenced by restricted gene flow resulting from isolation by distance due to habitat fragmentation. In total, we identified 102 outlier loci and annotated 455 candidate genes associated with the genomic basis of climate adaptation, which were enriched in functional categories related to development processes and stress response; both temperature and precipitation are important factors driving adaptive variation. In addition to single-nucleotide polymorphisms (SNPs), a total of 27 single-methylation variants (SMVs) had significant correlation with at least one of four climatic variables and 16 SMVs were located in or adjacent to genes, several of which were predicted to be involved in plant response to abiotic or biotic stress. Gradient forest analysis indicated that the central/east populations were predicted to be at higher risk of future population maladaptation under climate change. Our results demonstrate that local climate factors impose strong selection pressures and lead to local adaptation. Such information adds to our understanding of adaptive mechanisms to variable climates revealed by both population genome and epigenome analysis.

## Introduction

Global climate change is accelerating the melting of glaciers and sea level rise, which will have far-reaching and extensive impacts on the earth’s ecosystem. In response to climate change, organisms may migrate to higher latitude and altitude areas due to the gradual loss of their original conditions, which may eventually lead to species extinction [1–3]. The effects of global climate change on animals and plants have received much attention from evolutionary biologists [4–7]. During long-term evolutionary processes, organisms gradually adapt to external climate change, while many have unique mechanisms to adapt to rapid climate change. For example, local adaptation to variable climatic conditions (i.e. local genotypes have higher fitness than those of non-local genotypes) is critical for species persistence in response to climate change [8]. Studies focusing on local adaptation are of great significance for understanding how species evolve and differentiate while forecasting the impacts of global climate change on species distribution. Therefore, in the context of global climate change, there are a growing number of studies on genetic adaptation to abiotic selective pressures such as climate, including theoretical and empirical studies [8–10].

The process of local adaptation results from interactions of different evolutionary forces, including selection, genetic drift, mutation, and migration [8]. Knowledge of the population demographics of species will help us better understand the mechanisms of local adaptation. Moreover, species are sensitive to diverse selective pressures in spatially heterogeneous environments, which drives the emergence of local adaptation. Abiotic factors such as climatic and soil conditions, as well as biotic factors including species competition and animal herbivory, are driving factors for local adaptation in plants [11]. Among possible factors, temperature and precipitation are two important factors that produce the clearest signals of divergent selection across a variety of plant species [12, 13]. To discover genetic determinants of local adaptation, a major challenge is to identify outlier loci potentially subject to spatially divergent selection [8, 9].

Recently, improvements in high-throughput sequencing technologies and bioinformatic approaches make it feasible to detect genome-wide genetic variation and reveal the genomic properties associated with the process of local adaptation. Restriction-site associated DNA sequencing (RADseq), one of the high-throughput sequencing methods with restriction enzyme-based reduced representation of individual genomes, can dramatically reduce sequencing costs and produce tens of thousands of genetic markers in non-model species, making this approach widely used in ecological genomics studies [14]. Furthermore, combining genome scan approaches, which identify selection loci possessing significantly high genetic differentiation, and landscape genomic methods, which detect outlier loci associated with population-specific environmental covariables by genome–environment association (GEA), can enhance our understanding of the molecular bases of local adaptation [7, 8].

Epigenetic processes, as well as genetic variation, may alter phenotypes to improve plant adaptation to divergent environments [15]. Recent studies have highlighted the important role of epigenetic processes as a mechanism resulting in local adaptation. For example, DNA methylation was an important mechanism of local adaptation of natural oak populations to their environmental changes, as shown by Gugger *et al*. [16]. Therefore, studying population DNA methylation provides another perspective for understanding the molecular basis of local adaptation [9, 17]. Bisulfite-converted restriction site-associated DNA sequencing (bsRADseq), a method for quantifying the level of DNA methylation at the genomic level, can be useful for detecting evidence of epigenetic processes involved in local adaptation in the genomic context [17].

In addition, predicting the vulnerability of extant populations to future environmental changes at the genomic level enhances our ability to develop conservation measures for threatened species. Gradient forest (GF) models, which apply a machine-learning algorithm and account for non-linear associations of environmental and allelic variables [18], have been widely implemented in assessing species vulnerability to environmental change. GF models can identify populations with high genomic mismatch by integrating local adaptation information and environmental vulnerability models [19]. The combination of linear and non-linear regression models allows better prediction of the ability of a given species to adapt to future climate change [18].

**Table 1:** Detailed information for sampling sites, sample sizes (*n*) and genetic diversity of the 13 *A. eriantha* populations.

Population code	Location	Longitude (E)	Latitude (N)	Altitude (m)	Sample size	*π*	*H*_O_	*F*_IS_
WH	Wuhua County, Guangdong Province	115°23′	23°52′	686	12	.074	.081	−.009
LY	Luoyuan County, Fujian Province	119°24′	26°27′	529	12	.072	.073	−.001
LiS	Lishui city, Zhejiang Province	119°46′	28°15′	365	12	.073	.073	.002
SQ	Mount Sanqing, Jiangxi Province	118°03′	28°12′	589	12	.056	.060	−.005
RY	Ruyuan County, Guangdong Province	113°03′	24°57′	820	12	.067	.070	−.004
LS	Mount Lu, Jiangxi Province	115°58′	29°33′	1080	12	.067	.073	−.01
LC	Lichuan County, Jiangxi Province	116°50′	27°05′	277	12	.076	.077	0
DK	Dongkou County, Hunan Province	110°40′	27°14′	535	12	.042	.046	−.004
YP	Yuping County, Guizhou Province	108°52′	27°09′	506	12	.053	.040	.041
QY	Qiyang County, Hunan Province	112°06′	26°15′	146	12	.051	.052	−.003
GD	Guiding County, Guizhou Province	107°03′	26°15	1113	8	.026	.036	−.011
WGS	Mount Wugong, Jiangxi Province	114°13′	27°29′	619	12	.065	.070	−.005
HA	Hua’an County, Fujian Province	117°26′	24°52′	861	12	.075	.081	−.005

In this study we sought to reveal signatures of divergence in wild populations of *Actinidia eriantha* Benth., a species closely related to cultivated kiwifruit (*Actinidia chinensis* Planch.) [20, 21], using RADseq. We used two methods to identify the genetic features underlying local adaptation. First, we used a population genomics approach to identify outlier loci that are either under selection or linked to selected loci through ‘genetic hitchhiking’ [22]. Second, a landscape genomics approach was applied to establish the association between genomic regions and environmental factors in natural populations [23]. We detected evidence of DNA methylation involved in local adaptation of natural populations of kiwifruit using bsRADseq. Additionally, we used GF models to detect genomic mismatch between current and future climate scenarios. Knowledge of the loci underlying local adaptation and the vulnerability of populations to climate change provides valuable information to help formulate appropriate breeding and conservation strategies for this economically important plant, as well as helping to reveal the mechanisms of genetic response to climate change [24].

## Results

### RADseq data quality and processing

A total of 2400.88 M clean reads, representing ∼352.68 Gb of sequencing data, were obtained after the removal of low-quality sequences, sequences with ambiguous barcodes, and orphaned paired-end reads. The mean data size for samples was 2.32 Gb with an average read depth of 20.9× (range: 8.3–34.3×; Supplementary Data Table S1). After alignment to the *A. eriantha* reference genome and filtering, a total of 268 353 RAD loci containing 161 927 variant sites remained for subsequent analyses.

### Genetic diversity and structure

**Figure 1:**
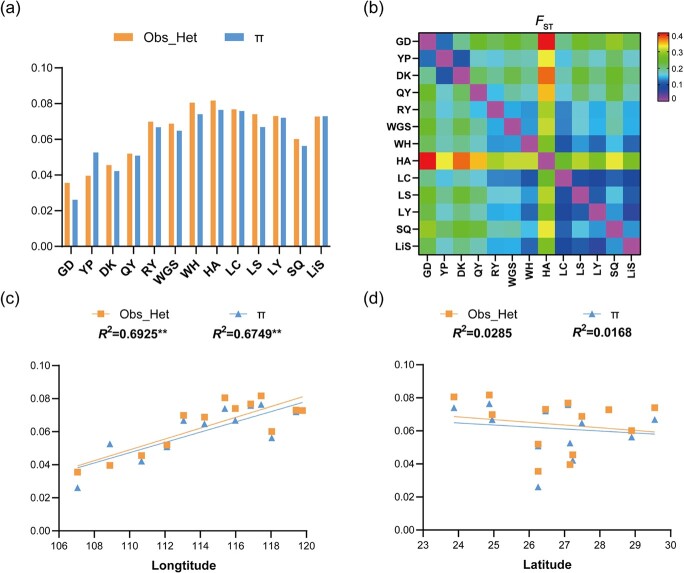
Population genetic variation and structure of natural populations of *A. eriantha* based on RADseq. **a** Observed heterozygosity (*H*_O_) and nucleotide diversity (*π*) for each population. **b** Pairwise genetic differentiation. Warmer colors indicate areas with a higher level of genetic differentiation. **c** Relationships between absolute longitude and genetic diversity. **d** Relationships between absolute latitude and genetic diversity.

Within each population, nucleotide diversity (*π*) ranged from 0.026 (GD) to 0.075 (HA). The observed heterozygosity (*H*_O_) was .016–.081 (Table 1; Fig. 1a). The global estimate of population differentiation (*F*_ST_) was 0.193 (*P* < .001). Average pairwise *F*_ST_ values across all populations ranged from 0.066 between LY and LiS populations to 0.415 between GD and HA populations (Fig. 1b; Supplementary Data Table S2). The observed heterozygosity and nucleotide diversity were significantly correlated with longitude but not with latitude (Fig. 1c and d). In general, the genetic diversity of the eastern populations was higher than that of the western populations.

**Figure 2:**
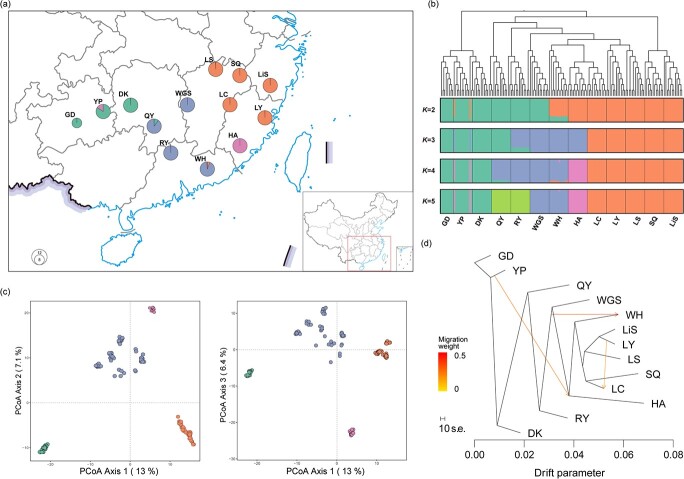
Genetic structure of *A. eriantha* based on RADseq. **a** Geographical location of the 13 sampling populations and their color-coded grouping based on fastSTRUCTURE analysis. **b** Population structure inferred by fastSTRUCTURE analysis (*K* = 2-5) and unrooted NJ tree of *A. eriantha* individuals. **c** PCoA for all populations. **d** Gene flow between *A. eriantha* populations detected by Treemix. Lines show gene flow, with arrows denoting its direction. Migration events are colored according to their weight: red denotes a high level of gene flow while yellow shows a low level of gene flow.

fastSTRUCTURE indicated that the 13 populations clustered into four groups (*K* = 4; Fig. 2a and b). These clusters correspond well to geographic regions, which were also supported by the principal coordinate analysis (PCoA) results (Fig. 2c). Along the first axis, all populations formed three different clusters (East cluster, Central cluster and West cluster). Along the second axis, the East cluster was further subdivided into two groups in which population HA was greatly differentiated from all other populations in the East cluster (Fig. 2c). The patterns of population structure shown in the neighbor-joining (NJ) tree were highly congruent to the patterns revealed in the PCoA analysis (Fig. 2b).

Isolation by distance (IBD) and isolation by environment (IBE) analyses were performed using Mantel tests. We detected significant IBD across all samples (*r* = .407, *P* = .018). The partial Mantel test revealed no evidence of IBE (*r*_E_ = .298, *P* = .086, Supplementary Data Fig. S1). In addition, Mantel tests between genetic distance and each bioclimatic distance, as well as between geographic distance and each bioclimatic distance, were all non-significant (Supplementary Data Table S3).

Three migration events between neighboring populations were revealed by Treemix. In addition, one migration event occurred between the relatively distant populations (HA and YP), suggesting an ancient long-distance dispersal event may have contributed to population structure (Fig. 2d). The three migration events were also confirmed by the Patterson’s *D* statistic (Patterson’s *D* > 0, *P* < .05), although the test between the LY and LC populations had a *Z*-score <3 (Supplementary Data Table S4).

### Outlier detection

Outlier tests using BayeScan identified 463 outlier loci that were putatively under selection (Supplementary Data Fig. S2). With a confidence level set to 99%, the hierarchical FDIST analysis identified 835 loci putatively under selection. Of the 835 outlier loci found in FDIST, 240 were also identified by BayeScan (Supplementary Data Fig. S3).

**Figure 3:**
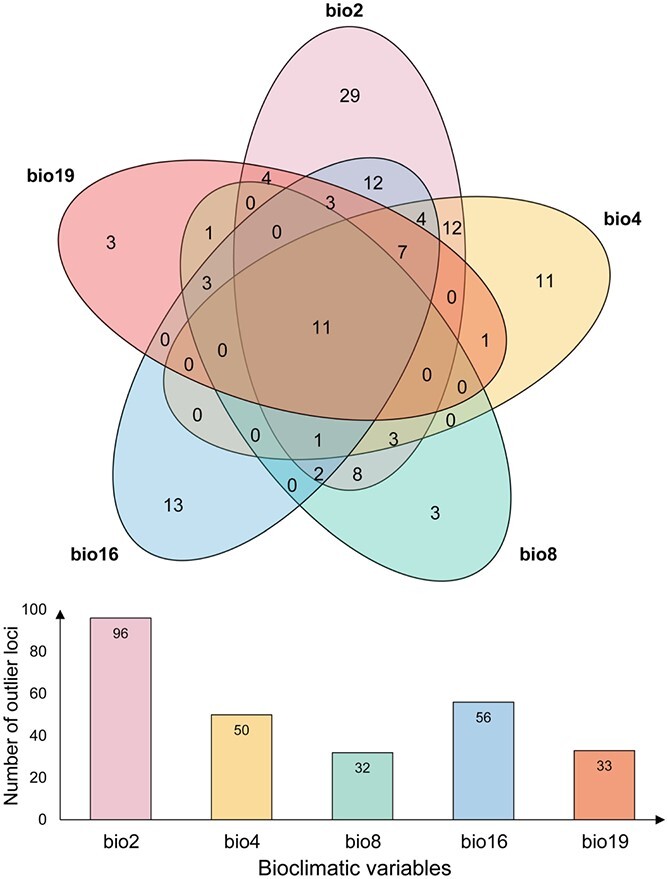
Venn diagrams illustrating the overlap of the 106 annotated outliers for each climatic variable.

With the *q*-value threshold of .05, the BayeScEnv analysis identified 131 unique loci correlated with at least one climatic variable (Supplementary Data Fig. S4), in which 96 were correlated with mean diurnal range (BIO2), 50 were associated with temperature seasonality (BIO4), 32 were correlated with mean temperature of the wettest quarter (BIO8), 56 were correlated with precipitation of the wettest quarter (BIO16), and 33 were correlated with precipitation of the coldest quarter (BIO19) (Fig. 3). A total of 1061 loci were identified using three different approaches, with 102 of the 1061 (9.6%) candidate loci being identified by all three methods (Supplementary Data Fig. S3). In addition, due to the relatively high *F*_ST_ of population HA compared with the other populations (Fig. 1b), we ran an additional BayeScEnv analysis after removing this population. The results showed that most of the detected environment-associated outliers overlapped with our initial analysis (Supplementary Data Fig. S5).

**Figure 4:**
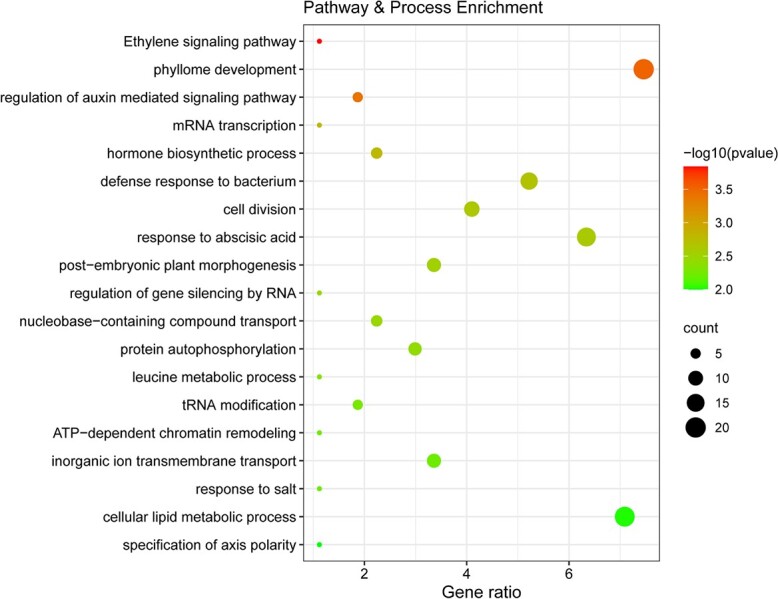
GO term enrichment for candidate genes.

Gene ontology (GO) enrichment analysis found biological processes that were enriched for candidate single-nucleotide polymorphisms (SNPs). By searching loci overlapping or within 10 kb of an exon, which is the distance that linkage disequilibrium plateaus in our sequencing data (Supplementary Data Fig. S6), a total of 455 protein genes that are physically linked to 102 outlier loci were identified to be under divergent selection (Supplementary Data Table S5). These genes were further classified with GO terms such as ‘ethylene signaling pathway’, ‘phyllome development’, ‘regulation of auxin mediated signaling pathway’, and ‘mRNA transcription’ (Fig. 4). Notably, some SNPs are located in candidate genes with functions associated with particular climatic variables (Table 2). The distributions of 455 protein genes on 29 chromosomes are shown in Supplementary Data Fig. S7. The number of candidate genes for each chromosome ranged from 3 to 41.

### bsRADseq data

The average number of clean reads per sample was 67.7 × 10^6^, with an average read depth of 10× (Supplementary Data Table S6). The average methylation across all samples in CpG, CHG, and CHH contexts were 25.3, 13.4 and 4.6%, respectively (Supplementary Data Table S6; Supplementary Data Fig. S8). A total of 1 176 162 single-methylation variants (SMVs) were identified after filtering SMVs with >10% missing data and <10% minimum range of variation (the difference between the maximum and minimum methylation per position is at least 10%). Based on the linear mixed model, 27 SMVs showed significant association with climatic factors after correcting for spurious association due to population structure. Of these 27 SMVs, 4 showed significant association with temperature seasonality (BIO4), and one with mean temperature of the wettest quarter (BIO8). A total of 12 out of 27 SMVs had significant correlation with precipitation of the wettest quarter (BIO16) and two with precipitation of the coldest quarter (BIO19). There were no SMVs associated with mean diurnal range (BIO2) (Supplementary Data Table S7). A total of 16 candidate genes were identified by proximity to an EWAS (epigenome-wide association study) signal. Among these, several genes have been reported to play a role in plant development, environmental adaptation or stress response (Table 3).

### Population-level vulnerability to climate change

A GF analysis was used to calculate the genomic offset of the 13 populations of *A. eriantha* based on all SNPs. The overall importance plot of the GF results revealed that precipitation of the wettest quarter (BIO16) and precipitation of the coldest quarter (BIO19) were the most important bioclimatic variables (Supplementary Data Fig. S9). The allelic turnover of 102 environment-associated loci detected by BayeScEnv was also assessed by cumulative importance in the GF results (*R*^2^ > 0). Most of the environment-associated loci (73/102) showed significant allelic turnover with five bioclimatic variables, of which 73 and 71 loci showed significant allelic turnover with BIO19 and BIO16, respectively (Supplementary Data Table S8).

**Figure 5:**
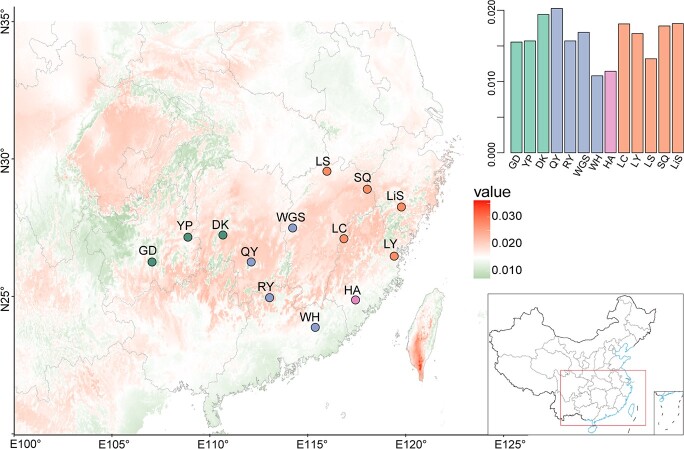
Predicted genomic offset across *A. eriantha* distribution under RCP8.5 scenarios in 2061–80 of the GCM-CC model. The color scale from green to red refers to increasing values of genetic offset. The bar chart of each panel shows estimated genetic offset for each sample location. Color-coding in the bar chart and points on the map correspond to the population structure defined in *A. eriantha*.

Under different representative concentration pathways (RCPs; RCP4.5, 6.0, and 8.5 of 2061–80) of two global climate models (GCMs; GCM-AC and GCM-CC), genomic offset to climate change was predicted to be higher from central/east populations compared with those from the mountains in the south and west (Fig. 5; Supplementary Data Fig. S10). Despite differences in estimated values of genomic offset under different future climatic scenarios, an increasing trend with the increase in emissions was observed (Supplementary Data Fig. S11). Central/east populations with the highest mismatch may be most maladapted to future climate change, potentially leading to regional declines or local extirpations. In addition, forward genetic offset (i.e. migration load) was estimated by estimating the minimum predicted offset. This showed a similar geographic pattern to local genetic offset, with higher migration loads in the central/east regions (Supplementary Data Fig. S12). Furthermore, the estimation of genetic offset using outlier SNPs showed a higher level of offsets under climate change, expanding to western populations (Supplementary Data Fig. S13).

**Table 2:** Summary of candidate genes jointly identified in this study and other studies.

SNP	Gene function	Climatic variable	*E*-value	Reference
MHT.Chr.201809044	Calcium-dependent protein kinase 4	BIO2	6.4E−49	[84]
MHT.Chr.201809045	Calcium-dependent protein kinase 17	BIO2	1.65E−34	[84]
MHT.Chr.201830014	ATP synthase subunit alpha	BIO2	2.25E−46	[39]
MHT.Chr.201826721	Cellulose synthase-like protein E6	BIO2	1.09E−59	[39]
MHT.Chr.201826722	Cellulose synthase-like protein E2	BIO2	4.31E−23	[39]
MHT.Chr.201824820	Ribosomal protein L18a-like protein	BIO2	1.93E−34	[29]
MHT.Chr.201832018	Late embryogenesis abundant protein 31	BIO2	1.23E−27	[85]
MHT.Chr.201813520	Probable serine/threonine-protein kinase	BIO2	0	[85, 86]
MHT.Chr.201823271	ABC transporter B family member 9	BIO4	0	[46]
MHT.Chr.201808827	Transcription factor MYB	BIO16	2.88E−123	[46]
MHT.Chr.201809163	Plant UBX domain-containing protein 8	BIO2	5.7E−152	[46]
MHT.Chr.201815640	PAP29 – purple acid phosphatase 29	BIO8	3.16E−178	[87]
MHT.Chr.201811317	NAC transcription factor	BIO2	1.49E−112	[45]
MHT.Chr.201812099	Histone-lysine *N*-methyltransferase	BIO16	0	[88]
MHT.Chr.201818777	Heat shock protein	BIO16	1.51E−08	[89]
MHT.Chr.201808355	Galacturonosyltransferase	BIO16	0	[86]
MHT.Chr.201836965	Alpha-1,3-arabinosyltransferase	BIO16	2.28E−37	[89]
MHT.Chr.201810028	Ethylene-responsive transcription factor	BIO2	1.47E−06	[88]
MHT.Chr.201826018	bZIP transcription factor 29	BIO16	0	[90]
MHT.Chr.201806938	Pentatricopeptide repeat-containing protein	BIO19	1.65E−09	[91]
MHT.Chr.201811857	Probable flavin containing monooxygenase 1	BIO2	0	[92]

**Table 3:** Annotation information on 16 genes with significant climate association based on bsRADseq.

Location	Climatic association	Closest gene via BLASTx match and function	BLASTx *P*-value	Reference
scaf_37.7157605	BIO4	*REV7*. DNA polymerase zeta processivity subunit [*Arabidopsis thaliana*]; tolerating exposure to DNA-damaging agents	4.60E−97	[93]
scaf_123.886806	BIO4	***UBP12***. Ubiquitin C-terminal hydrolase 12 [*A. thaliana*]; dehydration stress response; flowering	0	[48, 49]
scaf_147.2178690	BIO8	*ATXR5*. Probable histone-lysine *N*-methyltransferase ATXR5 [*Ricinus communis*]; plant development	0	[94]
scaf_111.15655300	BIO16	*PID*. Protein kinase PINOID [*A. thaliana*]; flower and root development, regulation of auxin signaling	0	[95]
scaf_147.281433	BIO16	***EF112***. Ethylene-responsive transcription factor ERF112 [*A. thaliana*]; pathogenesis stress, signal transduction	3.16E−34	[96]
scaf_55.488080	BIO16	*FBL4*. F-box/LRR-repeat protein 4 [*A. thaliana*]; catabolic process	2.79E−18	[97]
scaf_102.1291318	BIO16	***SMG7***. Nonsense-mediated mRNA decay factor SMG7 [*A. thaliana*]; growth and development, plant defense, hormone response	0	[98]
scaf_128.868076	BIO16	*SAMBA*. Protein SAMBA [*A. thaliana*]; cell proliferation during early development	6.24E−29	[99]
scaf_66.1377892	BIO16	*ALMT9*. Aluminum-activated malate transporter 9 [*A. thaliana*]; vacuolar malate channel	0	[100]
scaf_111.7222183	BIO19	*KEA4*. K(+) efflux antiporter 4 [*A. thaliana*]; K+ homeostasis; osmotic adjustment	6.78E−40	[101]
scaf_68.891676	BIO19	*SPY*. UDP-*N*-acetylglucosamine–peptide *N*-acetylglucosaminyltransferase SPINDLY [*Petunia hybrida*]; flowering time	0	[50]
scaf_14.77140	BIO19	*IPYR3*. Soluble inorganic pyrophosphatase 3 [*A. thaliana*]; controlling cellular levels of inorganic pyrophosphate	5.34E−142	[102]
scaf_26.413127	BIO19	***YSL6***. Probable metal-nicotianamine transporter YSL6 [*A. thaliana*]; metal stress response	0	[50]
scaf_68.1602078	BIO19	***PUB52***. U-box domain-containing protein 52 [*A. thaliana*]; salt, cold, and drought stresses	1.85E−145	[47]
scaf_28.5130564	BIO19	***OEP7***. Outer envelope membrane protein 7 [*A. thaliana*]; **salt stress**	3.30E−07	[103]
scaf_102.1573736	BIO19	*PPH*. Pheophytinase chloroplastic [*A. thaliana*]; leaf senescence	0	[104]

Genes involved in plant response to abiotic or biotic stress are shown in bold.

## Discussion

### Large-scale population structure

Although previous phylogeographic studies utilizing chloroplast and nuclear microsatellite markers established that the western areas of *A. eriantha* were colonized by westward expansion from the main refugia located at the eastern margin range of the species’ distribution, there was no clear east–west colonization route [25]. In the present study, a continuous loss of genetic diversity across the geographic distribution from east to west was detected based on the correlation between genetic diversity and longitude (Fig. 1c). The presented SNP data span a large portion of the genome with considerably more resolution than previous studies, supporting the hypothesis of east–west stepwise colonization. Genetic drift due to a series of founder events could consecutively reduce genetic variation during range expansion in *A. eriantha*, with the western populations maintaining the lowest diversity compared with the eastern populations. Accumulating evidence shows that the decline of heterozygosity along the east–west axis of range expansion has been found in other plant taxa [26, 27].

Four groups were revealed in *A. eriantha* by the Structure and PCoA analyses corresponding to four distinct geographic regions, which suggests that the population structure has been geographically shaped. Although no clear geographic barriers (i.e. lakes or mountains) separate the four clusters, a significant correlation between genetic and geographic distance was observed, suggesting the existence of barriers to gene flow between populations. *Actinidia eriantha* has experienced a rapid population decline, primarily due to increasing anthropogenic pressures (e.g. destructive harvesting and deforested-land reclamation) in natural habitats [28]. Current habitat fragmentation may augment isolation, reducing connectivity between populations and impeding genetic exchange among highly fragmented populations. The restricted gene flow among populations was also evident from the Treemix results, suggesting that gene flow between neighboring populations is scarce (Fig. 2d). Unexpectedly, several individuals of population YP (west cluster) share common genotypes with eastern populations (Fig. 2a). This shared ancestry suggests long-distance dispersal in the demographic history of the species. The long-distance dispersal events were also supported by a previous study, in which the western group shared common chloroplast haplotypes with the eastern group [25].

### Signatures of natural selection

The identification of outlier loci depends heavily on genetic models. The three analysis methods used here based on different models displayed large discrepancies in the identity and number of outlier loci. The number of outliers identified by BayeScan was approximately two times less than the values identified with FDIST, while BayeScEnv detected the minimum number of outliers. The lack of congruence amongst different methods has been reported in other studies [29]. Factors such as background selection, population structure, heterogeneous mutation and recombination rates, and past demographic events potentially result in a high false-positive rate in outlier detection [26, 30, 31]. In the present study, genetic drift is likely to have occurred during population expansion from east to west, producing clinal variation that is similar to patterns expected under positive selection. Additionally, the presence of population structure in our study may also result in correlated allele frequencies, which produce an excess of false-positive signal of selective sweeps in genome scans [26]. Currently, the common drawbacks of population genomic studies appear negligible [30]. To minimize the detection of false positives, combining the results from different methods may be an efficient way in which loci detected as selective outliers are likely to be true [32, 33]. In the present study, we employed three different algorithms (FDIST, BayeScan, and BayeScEnv) to detect outliers (Supplementary Data Fig. S3). Most outliers (78%) identified by BayeScEnv were also detected by the other two methods, suggesting BayeScEnv has a suitable control of false-discovery rate. Moreover, most of the outliers identified by BayeScEnv, both including the HA population and excluding the HA population, overlapped (Supplementary Data Fig. S5), suggesting the method of BayeScEnv has the advantage of reducing the effect of population structure on the outlier analysis.

A total of 102 outlier loci identified by the three methods were inferred to be under diversifying selection. These outlier loci were correlated with temperature or precipitation variables, indicating the importance of these variables in determining local adaptation. Outlier loci with high correlation to temperature or precipitation have been widely reported in plants such as *Cephalotaxus oliveri* [34], *Metrosideros polymorpha* [35], and *Eucalyptus melliodora* [36]. Temperature and precipitation are two important factors driving selective change along an environmental gradient [23, 37], and such a result is consistent with our current understanding of kiwifruit natural history and ecology. *Actinidia eriantha* grows best in relatively warm, wet and sheltered areas, with dry, cold environments being unsuitable [38]. Therefore, temperature and precipitation are two important limiting factors for the geographic distribution of kiwifruit species.

The 102 loci identified from *F*_ST_ outlier analysis and the GEA intersect with 455 candidate protein-coding genes within 10 kb (Supplementary Data Table S5). In line with findings from other plant species, climate adaptation in *A. eriantha* appears to be a genome-wide phenomenon [27, 39]. Selection on multiple genes may lead to local adaptation in *A. eriantha* populations. The majority of genes associated with climatic variables were associated with temperature (400/455), while the genes associated with precipitation were not as numerous (241/455). Some genes were associated with both temperature and precipitation variables (Fig. 3), suggesting a polygenic architecture of locally varying selection (cf. polygenic adaptation) in *A. eriantha*. According to the results of the GO enrichment analysis, the top four highly differentiated GO terms are ‘ethylene signaling pathway’, ‘phyllome development’, ‘regulation of auxin mediated signaling pathway’, and ‘mRNA transcription’ (Fig. 4). For example, two ethylene-responsive transcription factors (BBM2, EF122) that play crucial roles in regulating plant development and abiotic stress responses were identified [40]. Peroxidase (*PER6*), an enzyme that catalyzes an oxidation reaction of specific substrates using hydrogen peroxide, has been shown to be differentially expressed in response to low temperature and drought stress [41, 42]. Other significantly associated genes include *LEA31* and *BZP29*, which were annotated to the late embryogenesis abundant (LEA) and bZIP-T gene family, respectively (Table 2). The gene *LEA31*, a LEA protein gene, confers tolerance to abiotic stress and appears to function in the regulation of responses to abscisic acid or low temperature [43]. The gene *BZIP29* encodes a basic leucine zipper-type protein and appears to be involved in an abscisic acid-dependent signal transduction pathway when *Arabidopsis thaliana* faces drought and high-salinity environments [44]. Moreover, other genes such as bHlH (basic helix–loop–helix; *bHlH30*, *bHlH80*, *bHlH95*, and *bHlH*111), MYB (*MYB17* and *MYB52*), and WRK (*WRK46*), NAC (*NAC56*) transcription factors revealed in our study may be involved in abiotic stress responses, as has been found in other studies [45, 46]. Overall, most genes exhibiting outlier SNPs were found in gene families whose functions are known to confer tolerance to multiple abiotic stresses in model species. This implies the role that climate factors play as a possible selective agent in fostering local adaptation of *A. eriantha*.

DNA methylation, an important epigenetic phenomenon, may play an important role in shaping phenotypes and contribute to local adaptation to the environment [9]. Although the number of associated loci may be underestimated due to the small sample size, environmental association analyses revealed a total of 27 SMVs that are significantly correlated with a specific climate variable and 16 annotated genes. Several genes found to be adjacent to significant climate-associated SMVs have been reported to be involved in plant response to various environmental stress or adaptation [47–51]. For instance, *PUP52*, a member of the U-box domain-containing proteins, may play important roles in plant cold, salt, and drought stress responses [47]. Ubiquitin-specific proteases (UGPs) play an essential role in regulatory processes, such as plant development and stress responses [48]. In the present study, we found a locus with high similarity to *UBP12*, which mediates transcription under dehydration stress in *Capsicum annuum* [49]. *YSL6*, a member of the Yellow Stripe1-Like family of transporter proteins in *A. thaliana*, plays an essential role in heavy metal stresses [50]. *SPY* acts as a negative regulator of gibberellin (GA) action in *A. thaliana* and plays a role in regulating flowering time in *Nicotiana tabacum* [51]. However, whether methylation at these loci alters gene expression in *A. eriantha* remains unknown. Nevertheless, our results provide initial evidence that DNA methylation plays a role in plant adaptation to their environment. Future experimental work will need to assess whether the significant climate-associated genes identified in the present study are the actual cause of fitness-related phenotypic change.

### Prediction of genomic offset to future climate change

Rapid climate change may outpace the adaptive capacity of plant populations and change their genetic composition [52]. Populations with high genetic offset (mismatch between current allele variation and projected climatic variables) are less likely to keep pace with future climate change, potentially causing population declines or extirpations. Thus, assessing species’ genetic offset to future climate change is of great importance for knowledge of the potential persistence risk of a given species and developing conservation strategies [53]. As a robust method to assess the potential genetic offset by comparing the current and future gene–environment associations, GF modeling has been widely used in a variety of species [19, 54, 55]. In the present study, we used a GF model to map the genomic variation and predict the impact of future climate change on local adaptation of kiwifruit wild populations.

Under different scenarios of future climate change, the measures of genomic adaptation predict that populations from the central to eastern areas of *A. eriantha*’s distribution have higher vulnerability compared with the western and southern areas. The estimates of mitigation load showed a similar genetic offset pattern, implying that there was no population that had the potential to locally grow better and preadapt to future climates in the eastern and central region. As expected, genomic offset increases under more severe climate change scenarios (Fig. 5; Supplementary Data Figs S10 and S11). Furthermore, genetic offsets estimated using outlier SNPs showed a pattern of increased genomic offset, suggesting that adaptive loci are more vulnerable to climate changes. Compared with the southwest mountain areas, anthropogenic activities are more frequent in central/east regions, implying the habitat here is more fragmented [28]. Overall, our data suggest that the central/east populations of *A. eriantha* are expected to have relatively low adaptive capacity and face a high risk of losing genetic diversity or climate-induced extinction in the context of future climate change and continuing habitat loss, indicating that these populations should be given priority for conservation.

In the present study, we found that precipitation measures are the strongest abiotic factors that correlate with allelic variation (Supplementary Data Fig. S9). The importance of precipitation in the evolution of kiwifruit species is consistent with other tree species [55]. According to the prediction of genetic offset, central/east populations of *A. eriantha* are expected to face drought conditions in the future and will be more sensitive to rainfall change, which is consistent with their high genomic offset. On the contrary, western and southern populations contain the lowest genomic offset and may be preadapted to future water stress scenarios. Moreover, compared with central/east populations, a lower migration load was detected in southern and western populations. Thus, assisted gene flow by transplantation (e.g. transplanting foreign genotypes from arid regions into vulnerable areas with a high likelihood of rainfall decline in GF analysis) or artificial seed transport from western and southern populations to central/east populations should be considered [56–58]. Notably, an ancient long dispersal event in the demographic history of *A. eriantha* was found, suggesting there is a potential to use assisted migration for conservation. Nonetheless, the evolutionary response to future climate change will be complex as adaptation is influenced by multiple fundamental processes of evolution, such as mutation, recombination, gene flow, and genetic drift [55]. More research about climatic tolerance of *A. eriantha* is therefore needed.

### Conclusions

Our study constitutes the most exhaustive study of genetic diversity and genetic structure for wild populations of kiwifruit species at genomic level. The key findings from this work are that *A. eriantha* is inferred to have an east–west colonization route and adaptive divergence among local populations. Combining genome scan and landscape genomic methods, 455 candidate genes were robustly detected, with the most prominent signatures of molecular selection among *A. eriantha* populations. The central/east populations were predicted to be more at risk of future population maladaptation under climate change, especially for adaptive variation. In addition, DNA methylation may play a role in local adaptation to climatic change for kiwifruit species, even though the actual role of individual candidate loci involved in local adaptation is still unclear. Further experimental studies, ideally involving fitness tests and gene function verification, is required to confirm the role of genes in local adaptation to climate. Reduced representation approaches such as RADseq, which only cover a small proportion of the genome, may not provide a sufficiently detailed picture of the genomic regions under selection. Further research will be required to gain more insights into which candidates covary with phenotypic variation using approaches such as genome resequencing.

## Materials and methods

### Study species and sampling

*Actinidia eriantha* (2*n* = 58) is functionally dioecious, with male and female individuals [59]. The species is a perennial climbing plant characterized by fruits densely covered by milk white to dark yellow hairs or tomenta [59]. *Actinidia eriantha* is an important economic species, which has been used in breeding programs to extend shelf life and increase vitamin C concentration of kiwifruits [28]. The species is abundant in subtropical zones of China, occurring in a wide range of habitats at an altitude of 200–1500 m. Seeds of *A. eriantha* are dispersed primarily by gravity and occasionally by frugivorous animals [28]. The roots of *A. eriantha* were used as the traditional Chinese medicine to treat breast carcinoma, gastric carcinoma, and hepatitis. Most adult individuals of *A. eriantha* in eastern China have been destroyed due to destructive harvesting (excavation of roots in adult trees).

A total of 152 individuals from 13 populations covering the distribution range of the species were collected (Table 1). Sample size varied between sites due to variation in collection success. For each site, sampled individuals were at least 100 m apart. All the sampled fresh leaves were immediately placed in silica gel and stored at room temperature.

### RADtag sequencing

Genomic DNA was extracted from 20 mg of dried leaves with a modified CTAB method [60]. The quality and quantity of DNA were determined by electrophoresis on 1.5% agarose gels. The RAD library construction followed the method of Zhang *et al*. [10]. Individual barcoded RAD samples were sent to BGI-Shenzhen (Shenzhen, Guangdong, China) for sequencing on an Illumina HiSeq 2000 instrument following a paired-end 150-bp protocol.

### Reduced-representation bisulfite sequencing

One individual from each site was used for bsRADseq. The bsRADseq libraries were prepared according to the protocol of Trucchi *et al*. [61]. Thirteen reduced-representation libraries were then amplified by 13 cycles of PCR with Illumina primers and sequenced on an Illumina HiSeq 2000 instrument at BGI-Shenzhen (Shenzhen, Guangdong, China) following a paired-end 150-bp protocol.

### Data analysis for RADseq

#### Data processing, assembly and alignment

To ensure high-quality genotype calls, reads lacking correct barcodes and containing >50% low-quality bases (Phred quality <20) were discarded by Trimmomatic v0.36 [62]. After trimming, reads shorter than 36 bp were also removed. The filtered reads were then aligned to the *A. eriantha* reference genome version 2.0 [63] using BWA v0.7.12 with a maximum mismatch of 5 [64]. Duplicate reads were discarded using Picard Tools v1.133 (http://broadinstitute.github.io/picard/). For each sample, alignments of all paired-end reads were converted to binary format and sorted using SAMtools v0.1.19 [65]. The reads around putative indels were corrected by local realignment using GATK v3.2.2 [66].

Several filtering steps involving the catalog of reads were performed to minimize SNP calling errors using the POPULATIONS module in the STACKS pipeline. SNPs showing an excess of heterozygotes and deviating from Hardy–Weinberg equilibrium were removed. SNPs with a missing genotype rate <20% and minimum allele frequency >0.01 across all sampled individuals were retained. Loci showing heterozygosity >.5 were removed to exclude the potential homologs. Linked SNPs were filtered using the – *write_single_snp* option in POPULATIONS. In addition, bi-allelic SNPs were retained when the distance between SNPs and any indels was >5 bp.

#### Genetic diversity and population structure

We estimated the genome-wide genetic diversity parameters nucleotide diversity (*π*), observed heterozygosity (*H*_O_), and inbreeding coefficient (*F*_IS_) using the POPULATIONS module in the STACKS pipeline. Pairwise population differentiation (*F*_ST_) values were estimated using the method of Weir and Cockerham [67] in the *hierfstat* package in R v3.6.1 [68].

For population structure analyses, we generated selectively neutral markers by removing detected outliers (1061 outliers) from the data set. Both Bayesian clustering and PCoA were used to estimate population structure. Bayesian clustering was performed in fastSTRUCTURE v1.0 [69] considering *K =* 2–10 ancestral clusters. The best *K* value was chosen using the *chooseK.py* function. A PCoA was performed using the *dartR* package (*gl.pcoa* function) to survey the existence of spatial segregation of genetic groups. Moreover, an NJ tree was constructed using MEGA X [70]. Confidence intervals were calculated with 1000 bootstrap samples.

IBD was evaluated by comparing linearized *F*_ST_ values [*F*_ST_/(1 − *F*_ST_)] with log-transformed geographic distance using a Mantel test with 10 000 permutations in the R package Vegan v2.6-2 [71]. Pairwise geographic distance was calculated as the shortest distance between sites. A partial Mantel test was used to determine IBE by regressing genetic distance matrices against pairwise environmental distances while controlling for log-transformed geographic distance using the R package Vegan v2.6-2 [71]. Environmental distances were calculated as Euclidian distances. A total of 19 bioclimatic variables recorded from 1970 to 2000 at a 2.5-minute arc resolution were extracted from WorldClim (http://www.diva-gis.org/climate) using DIVA-GIS v7.5 [72]. To avoid multicollinearity, five bioclimatic variables were selected based on correlation analysis (|*r*| < .8) using ENMTools v.1.3 [73]. The five climatic variables examined included mean diurnal range (BIO2), temperature seasonality (BIO4), mean temperature of wettest quarter (BIO8), precipitation of wettest quarter (BIO16), and precipitation of coldest quarter (BIO19). A principal component analysis (PCA) was conducted to reduce multicollinearity between bioclimatic variables using the R package Vegan. The first three principal components that explained >89% of the total variation were used to calculate environmental distance. In addition, we also ran Mantel tests between the selected five climatic variables (distance matrix in R) and genetic distance separately.

Treemix v1.171 [74] was used to infer gene flow between populations based on the frequency variation of SNPs using the default parameters and assuming 0–8 migration edges (‘-m’ = 0–8). The optimum number for m was identified using the R package OptM [74]. Patterson’s *D* statistic (ABBA-BABA test) was further used to verify the gene flow detected by Treemix using Dsuite v0.5 based on the minimum *D* (D_min_) score regardless of the tree topology [75]. In the ABBA-BABA test, P2 and P3 were the two target populations, and P1 was the one close-related to the target population (Supplementary Data Table S4). The pattern BBAA refers to P1 and P2 with the common derived allele, ABBA to P2 and P3 with the common derived allele, and BABA to P1 and P3 sharing the derived allele. The *Z*-scores calculated as *Z* = *D*/std_err (*D*) and the associated *P*-values were used to assess the significance of ABBA-BABA tests where *D* = (*n*ABBA − *n*BABA)/(*n*ABBA + *n*BABA).

#### Detection of loci under selection

Two alternative genome-scan methods were used to identify loci potentially targeted by selection. First, we used BayeScan v2.1, which implements a hierarchical Bayesian approach, to infer the posterior probability of each locus being under selection [32]. Outliers were calculated three times to ensure robustness. The parameters were set for a burn-in of 10 000 iterations, a thinning interval of 50, and a sample size of 10 000. Outliers were identified using the *q*-value cutoff of 1% significant false discovery rate (FDR). Second, we used an FDIST approach implemented in Arlequin v3.5 [76]. This method uses coalescent simulations under a hierarchical island model and provides an expected neutral distribution of *F*_ST_ value conditioned on heterozygosity. We ran FDIST with selective neutral SNPs detected by BayeScan first to establish a selectively neutral threshold (null distribution), and then reanalyzed with all SNPs. Loci outside of the null distribution were considered candidates for selection. Loci under selection show a higher value of *F*_ST_ than loci subjected only to gene flow and genetic drift.

Both BayeScan and FDIST cannot determine which climatic variables may be driving directional selection on loci. Associations between SNP allele frequencies and climate variables were calculated based on a Bayesian linear model method [77] to test whether *F*_ST_ increased with climatic differentiation, as implemented in BayeScEnv v1.1 with default settings [78]. One main advantage of the BayeScEnv method is the ability to correct for background levels of population structure and differences in sample size among populations. Five selected climate variables (BIO2, BIO4, BIO8, BIO16, BIO19) were standardized before analysis. We ran a separate BayeScEnv analysis for each variable. To search for outliers linked to a specific climate variable, we used the *q*-value for the model including the environmental factor (*q*val_g < .05).

#### SNP annotation and gene ontology analysis

LD decay analysis was performed by PopLDdecay v3.41 [79]. The decay of LD with physical distance between SNPs occurs at 10 kb, at which LD is reduced and reaches equilibrium. (Supplementary Data Fig. S6). To investigate the potential role of the candidate SNPs, we conducted annotation of the reference sequences using BLASTx searches against all available plant genomes with an E-value cut-off of 1 × 10^5^. Only SNPs that were closely linked to a mapped gene in the *A. eriantha* reference genome (overlapping or within 10 kb of an exon) were used. GO term enrichment for candidate genes was performed using the AgriGO toolkit v2.0 [80] based on Fisher’s exact test with Bonferroni correction (a *P*-value cut-off of .05).

#### Genomic offset analysis

Genomic offset under climate change is a predictive measure of the mismatch between contemporary and future spatial genomic composition [54]. GF is a machine-learning regression tree approach derived from a random forest algorithm that allows for handling non-linear correlations between allele frequencies and climate variables [18]. A GF model was used to infer population genomic offset under future climate change following the method of Fitzpatrick and Keller [19]. Future bioclimatic variables under RCP4.5, 6.0, and 8.5 scenarios of the GCM-CC climatic model as well as RCP4.5 and 8.5 scenarios of the GCM-AC climatic model were downloaded from WorldClim using the R package raster v3.6–3 (*getData* function). The GF analysis was conducted using the R package gradientForest v0.1–32 [18] following the method of Cao *et al.* [81]. Briefly, we first estimated allele frequencies and fitted them to current bioclimatic variables (ntree = 500, nbin201, corr.threshold = 0.5). Additionally, following the approach implemented in Sang *et al*. [82], we analyzed the forward genetic offset (migration load) by detecting the minimum predicted offset, assuming the specific extant population has the potential to migrate to any location in the distribution areas of *A. eriantha*. We also used outlier SNPs to estimate allele frequencies to predict genomic offset. The fitted model was then applied to calculate the Euclidian distance between genomic compositions under the contemporary and future projected climates. The distribution areas with higher values of Euclidian distances are predicted to have a larger genetic offset.

### Data analysis for bsRADseq

Reads were filtered using the same method as used for RADseq. Cytosine methylation in CG, CHG, and CHH contexts for each sample were measured by BS-SEEKER2 [83]. The degree of methylation at a site was quantified as a continuous variable. The SMVs were defined referring to the method of Gugger *et al*. [16]. To reveal the associations of epigenetic variation with climate, EWAS between SMVs and climate variables (BIO2, BIO4, BIO8, BIO16, BIO19) were performed using the linear mixed model as implemented in package pyLMM (https://github.com/nickFurlotte/pylmm), which offers the advantage of accounting for genetic structure and relatedness among individuals. The methylation levels were set as the explanatory variables and the environmental variables to the response variables. The methylation data were used to estimate the kinship matrix by referring to the method of Gugger *et al*. [16]. The model *y* = *μ* + *x*ꞵ + *g* + *e* was applied, where *μ* is a common intercept term, *x* is the corresponding design matrix based on methylation levels, ꞵ is the fixed effect, *g* is the polygenic background effect and *e* is a residual term, with Var(*g*) = *Kσ_g_*^2^ and Var(*e*) = *Iσ_e_*^2^, where *K* is the kinship matrix estimated according to the method of Gugger *et al*. [16] and *I* is the identity matrix. The significance thresholds were estimated by permutation testing. We performed permutation tests (with 200 repeats) by randomly selecting 10 000 SMVs for each iteration. Here, the permutated significance threshold for BIO2, BIO4, BIO8, BIO16, and BIO19 corresponded to *P*-values (corresponding to the level of .05) of 5.6E−6, 1.5E−6, 1.6E−5, 1.7E−6, and 9.0E−6, respectively. These values are approximately two to three orders of magnitude larger than the significance threshold obtained from Bonferroni correction (4.25E−8), which would give an overly stringent estimate of significance due to small sample size. Candidate loci identified in EWAS were searched for in the non-redundant Swiss-Prot protein database using a BLASTp analysis. All parameters were kept at their default values with a cut-off E-value <1.0E−5.

## Acknowledgements

We thank Na Wei for her assistance in data analysis. This study was funded by the National Natural Science Foundation of China (grants 31770374 and 32070377) and the Key Projects of the Joint Fund of the National Natural Science Foundation of China (grant U1802232).

## Author contributions

X.Y. and H.W. designed the project. X.Z. and F.L. performed data analysis. R.G., R.S., and Q.J. sampled the plant materials. X.Z., R.G., and X.Y. wrote the manuscript. J.B.L. revised the manuscript. All authors read and approved the final manuscript.

## Data availability

All RADseq clean reads and *bsRADseq* clean reads were deposited at NCBI under the bio-project accession numbers PRJNA788157 and PRJNA788205, respectively.

## Conflict of interest

The authors declare no competing interests.

## Supplementary data

Supplementary data is available at *Horticulture Research* online.

## Supplementary Material

Web_Material_uhad031
