# Supplementary material for: The genomic and epigenetic footprint of local adaptation to variable climates in kiwifruit
Source: Hortic Res. 2023 Feb 21;10(4):uhad031. doi: 10.1093/hr/uhad031 (PMC10548413; doi:10.1093/hr/uhad031)
Supplement: Web_Material_uhad031 [file web_material_uhad031.zip › Table S2.docx]

**Table S2** Paired population-level *F*_ST_ estimated by hierfstat using Weir and Cockerham's method.

| Population | GD | YP | QY | RY | LY | WGS | LS | LC | LiS | WH | HA | SQ | DK |
| --- | --- | --- | --- | --- | --- | --- | --- | --- | --- | --- | --- | --- | --- |
| GD | 0.0000 | 0.1219 | 0.2578 | 0.2172 | 0.2241 | 0.2533 | 0.2645 | 0.1987 | 0.2214 | 0.2166 | **0.4147** | 0.2931 | 0.1941 |
| YP | 0.1219 | 0.0000 | 0.1715 | 0.1599 | 0.1710 | 0.1845 | 0.1942 | 0.1554 | 0.1701 | 0.1787 | 0.3315 | 0.2125 | 0.1079 |
| QY | 0.2578 | 0.1715 | 0.0000 | 0.1508 | 0.1792 | 0.1868 | 0.2075 | 0.1622 | 0.1786 | 0.1730 | 0.3514 | 0.2287 | 0.2135 |
| RY | 0.2172 | 0.1599 | 0.1508 | 0.0000 | 0.1436 | 0.1422 | 0.1642 | 0.1300 | 0.1429 | 0.1356 | 0.3032 | 0.1805 | 0.1925 |
| LY | 0.2241 | 0.1710 | 0.1792 | 0.1436 | 0.0000 | 0.1449 | 0.1117 | 0.0777 | **0.0661** | 0.1178 | 0.2787 | 0.1354 | 0.2009 |
| WGS | 0.2533 | 0.1845 | 0.1868 | 0.1422 | 0.1449 | 0.0000 | 0.1652 | 0.1295 | 0.1442 | 0.1370 | 0.3162 | 0.1844 | 0.2231 |
| LS | 0.2645 | 0.1942 | 0.2075 | 0.1642 | 0.1117 | 0.1652 | 0.0000 | 0.1001 | 0.1117 | 0.1365 | 0.3093 | 0.1556 | 0.2331 |
| LC | 0.1987 | 0.1554 | 0.1622 | 0.1300 | 0.0777 | 0.1295 | 0.1001 | 0.0000 | 0.0799 | 0.1054 | 0.2602 | 0.1146 | 0.1807 |
| LiS | 0.2214 | 0.1701 | 0.1786 | 0.1429 | 0.0661 | 0.1442 | 0.1117 | 0.0799 | 0.0000 | 0.1177 | 0.2777 | 0.1349 | 0.1999 |
| WH | 0.2166 | 0.1787 | 0.1730 | 0.1356 | 0.1178 | 0.1370 | 0.1365 | 0.1054 | 0.1177 | 0.0000 | 0.2788 | 0.1518 | 0.1961 |
| HA | 0.4147 | 0.3315 | 0.3514 | 0.3032 | 0.2787 | 0.3162 | 0.3093 | 0.2602 | 0.2777 | 0.2788 | 0.0000 | 0.3288 | 0.3804 |
| SQ | 0.2931 | 0.2125 | 0.2287 | 0.1805 | 0.1354 | 0.1844 | 0.1556 | 0.1146 | 0.1349 | 0.1518 | 0.3288 | 0.0000 | 0.2548 |
| DK | 0.1941 | 0.1079 | 0.2135 | 0.1925 | 0.2009 | 0.2231 | 0.2331 | 0.1807 | 0.1999 | 0.1961 | 0.3804 | 0.2548 | 0.0000 |
