# Supplementary material for: The genomic and epigenetic footprint of local adaptation to variable climates in kiwifruit
Source: Hortic Res. 2023 Feb 21;10(4):uhad031. doi: 10.1093/hr/uhad031 (PMC10548413; doi:10.1093/hr/uhad031)
Supplement: Web_Material_uhad031 [file web_material_uhad031.zip › Table S3.docx]

**Table S3** Mantel tests between genetic distances and each bioclimatic distances as well as between geographic distances and bioclimatic distances.

| Bioclimate | Bioclimatic vs. genetic distances | | Bioclimatic vs. geographic distances | |
| --- | --- | --- | --- | --- |
|  | Mantel statistic *r* | *P* | Mantel statistic *r* | *P* |
| BIO2 | 0.0326 | 0.3608 | 0.0441 | 0.3184 |
| BIO4 | 0.2886 | 0.0962 | -0.0663 | 0.6748 |
| BIO8 | -0.0879 | 0.6625 | -0.0842 | 0.7403 |
| BIO16 | -0.0159 | 0.4541 | 0.1485 | 0.1162 |
| BIO19 | 0.2101 | 0.0985 | 0.1909 | 0.0736 |
