# Supplementary material for: The genomic and epigenetic footprint of local adaptation to variable climates in kiwifruit
Source: Hortic Res. 2023 Feb 21;10(4):uhad031. doi: 10.1093/hr/uhad031 (PMC10548413; doi:10.1093/hr/uhad031)
Supplement: Web_Material_uhad031 [file web_material_uhad031.zip › Table S4.docx]

**Table S4** The results of ABBA-BABA tests for potential gene flow between populations of *A. eriantha*

| **P1** | **P2** | **P3** | **D statistic** | **Z-score** | ***p*-value** | **f4-ratio** | **BBAA** | **ABBA** | **BABA** |
| --- | --- | --- | --- | --- | --- | --- | --- | --- | --- |
| GD | YP | HA | 0.117 | 6.513 | 7.376e-11 | 0.043 | 768.784 | 349.606 | 276.373 |
| LiS | LY | LC | 0.015 | 2.871 | 0.004 | 0.041 | 764.999 | 569.562 | 553.225 |
| RY | WGS | WH | 0.045 | 3.685 | 2.291e-4 | 0.062 | 693.945 | 545.607 | 498.266 |
