# Supplementary material for: The genomic and epigenetic footprint of local adaptation to variable climates in kiwifruit
Source: Hortic Res. 2023 Feb 21;10(4):uhad031. doi: 10.1093/hr/uhad031 (PMC10548413; doi:10.1093/hr/uhad031)
Supplement: Web_Material_uhad031 [file web_material_uhad031.zip › Table S6.docx]

**Table S5** Sequencing and alignment data of whole genome and Genomic methylation level of *Actinidia eriantha*.

| Population | Raw_reads | Raw_bases  (G) | Clean_reads | Clean_bases  (G) | BS conversion  rate (%) | mC percent  (%) | mCpG percent (%) | mCHG percent (%) | mCHH percent (%) |
| --- | --- | --- | --- | --- | --- | --- | --- | --- | --- |
| LC | 68111102 | 20.43 | 67918915 | 20.14 | 99.587 | 8% | 24.49% | 14.51% | 4.44% |
| LiS | 68003070 | 20.40 | 67781846 | 20.11 | 99.687 | 9.67% | 31.44% | 16.4% | 5.29% |
| WH | 67761529 | 20.33 | 67525240 | 20.03 | 99.674 | 8.9% | 25.95% | 14.32% | 5.44% |
| HA | 67701995 | 20.31 | 67522856 | 20.09 | 99.570 | 4.76% | 17.99% | 7.86% | 2.26% |
| YP | 67924482 | 20.38 | 67755182 | 20.16 | 99.635 | 7.78% | 22.59% | 12.55% | 4.77% |
| QY | 67712991 | 20.31 | 67462365 | 20.02 | 99.654 | 10.64% | 27.54% | 14.23% | 7.51% |
| GD | 67250657 | 20.18 | 67000909 | 19.82 | 99.656 | 7.04% | 22.38% | 11.97% | 3.92% |
| RY | 67764700 | 20.33 | 67534055 | 20.03 | 99.654 | 8.74% | 28.64% | 14.13% | 4.87% |
| LY | 67878202 | 20.36 | 67651376 | 20.08 | 99.580 | 9.75% | 30.7% | 15.95% | 5.58% |
| WGS | 67787959 | 20.34 | 67618697 | 20.13 | 99.684 | 7.29% | 23.68% | 12.77% | 3.93% |
| LS | 67956927 | 20.39 | 67800564 | 20.17 | 99.638 | 9.35% | 29.48% | 15.89% | 5.24% |
| DK | 68856252 | 20.66 | 68697599 | 20.44 | 99.626 | 6.09% | 20.48% | 10.94% | 3.12% |
| SQ | 68358303 | 20.51 | 68203297 | 20.29 | 99.595 | 6.47% | 23.55% | 12.38% | 2.92% |
| Average | 67928320 | 20.38 | 67728684 | 20.12 | 99.63% | 8.04% | 25.30% | 13.38% | 4.56% |

Note: BS conversion rate indicate the rate of C changed into T by bisulfite.
