# Supplementary figures and images for: The genomic and epigenetic footprint of local adaptation to variable climates in kiwifruit
Source: Hortic Res. 2023 Feb 21;10(4):uhad031. doi: 10.1093/hr/uhad031 (PMC10548413; doi:10.1093/hr/uhad031)

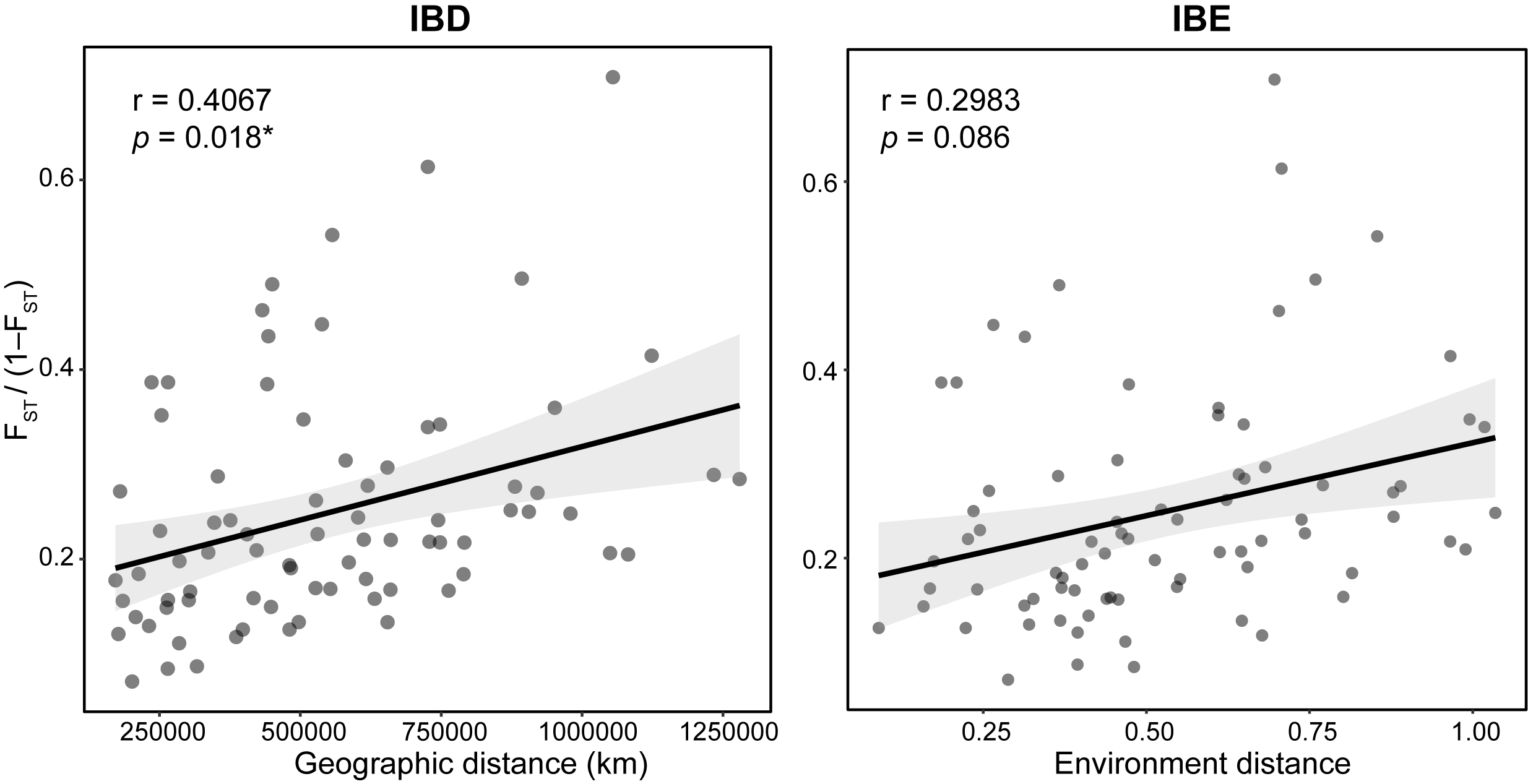

Supplement: Web_Material_uhad031 [file web_material_uhad031.zip › Fig S1.tif]

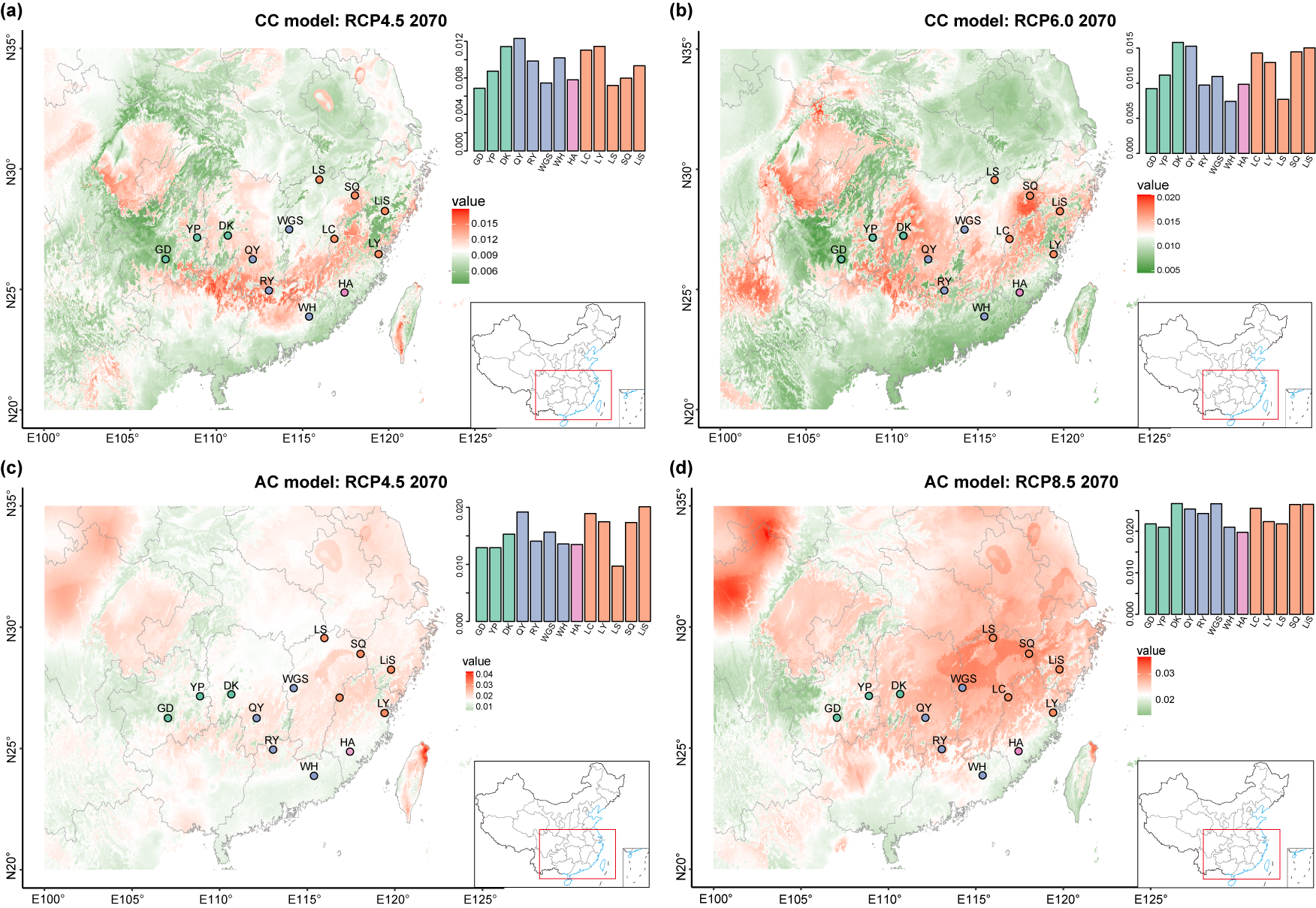

Supplement: Web_Material_uhad031 [file web_material_uhad031.zip › Fig S10_combinedc2.tif]

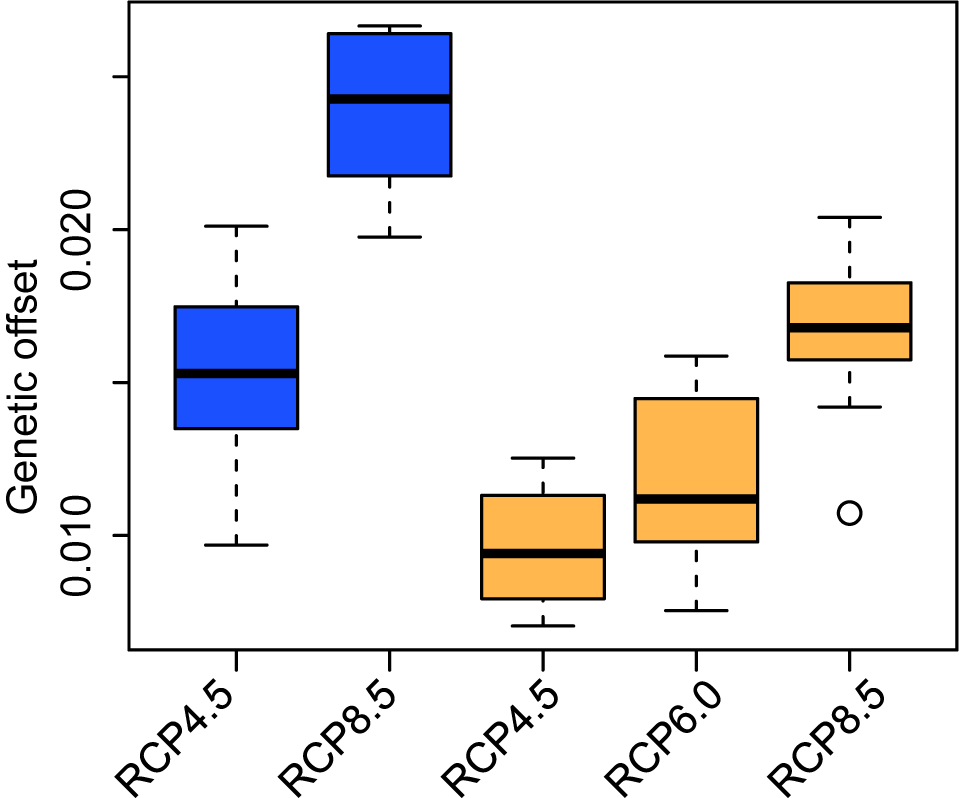

Supplement: Web_Material_uhad031 [file web_material_uhad031.zip › Fig S11_models.tif]

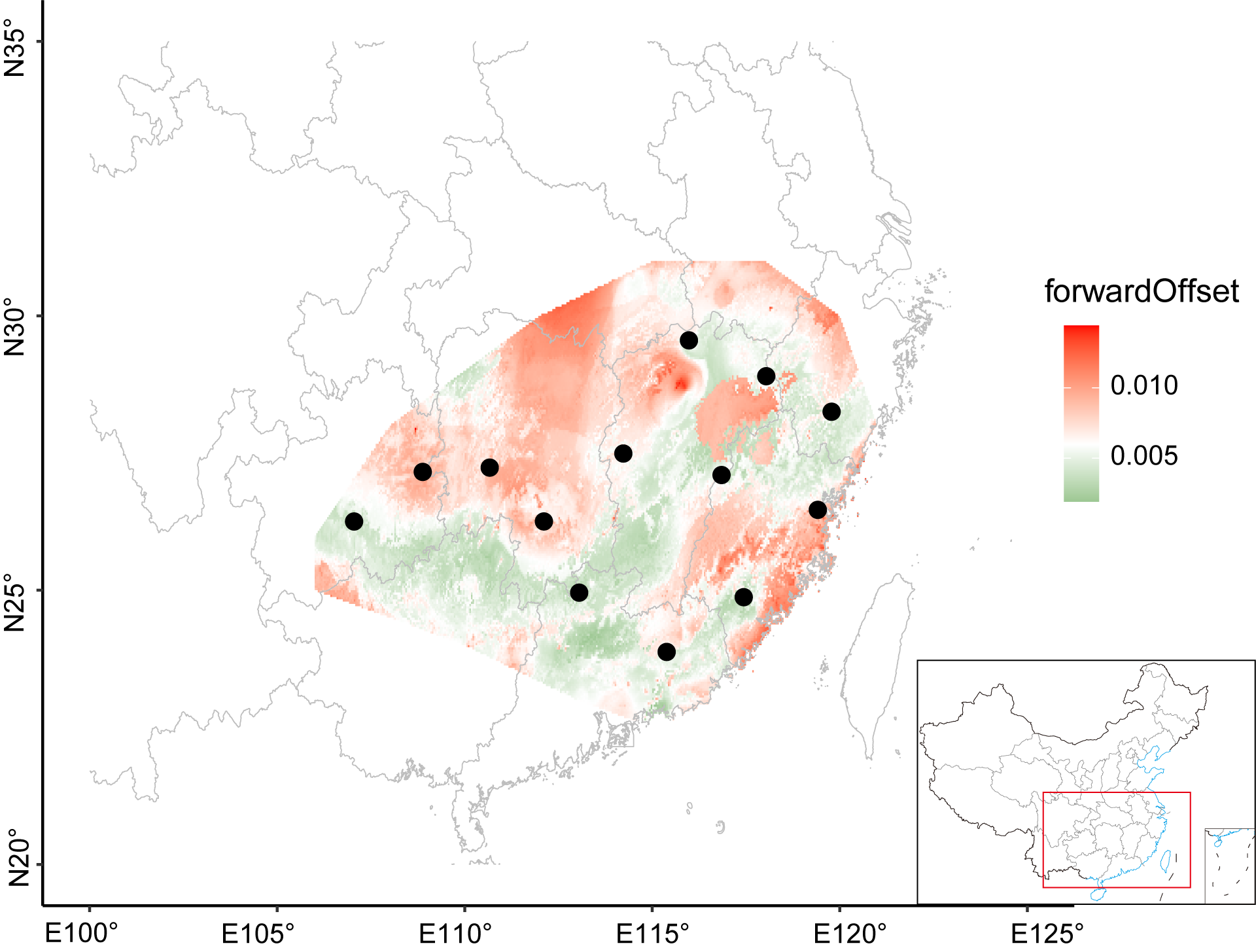

Supplement: Web_Material_uhad031 [file web_material_uhad031.zip › Fig S12_MLoad.tif]

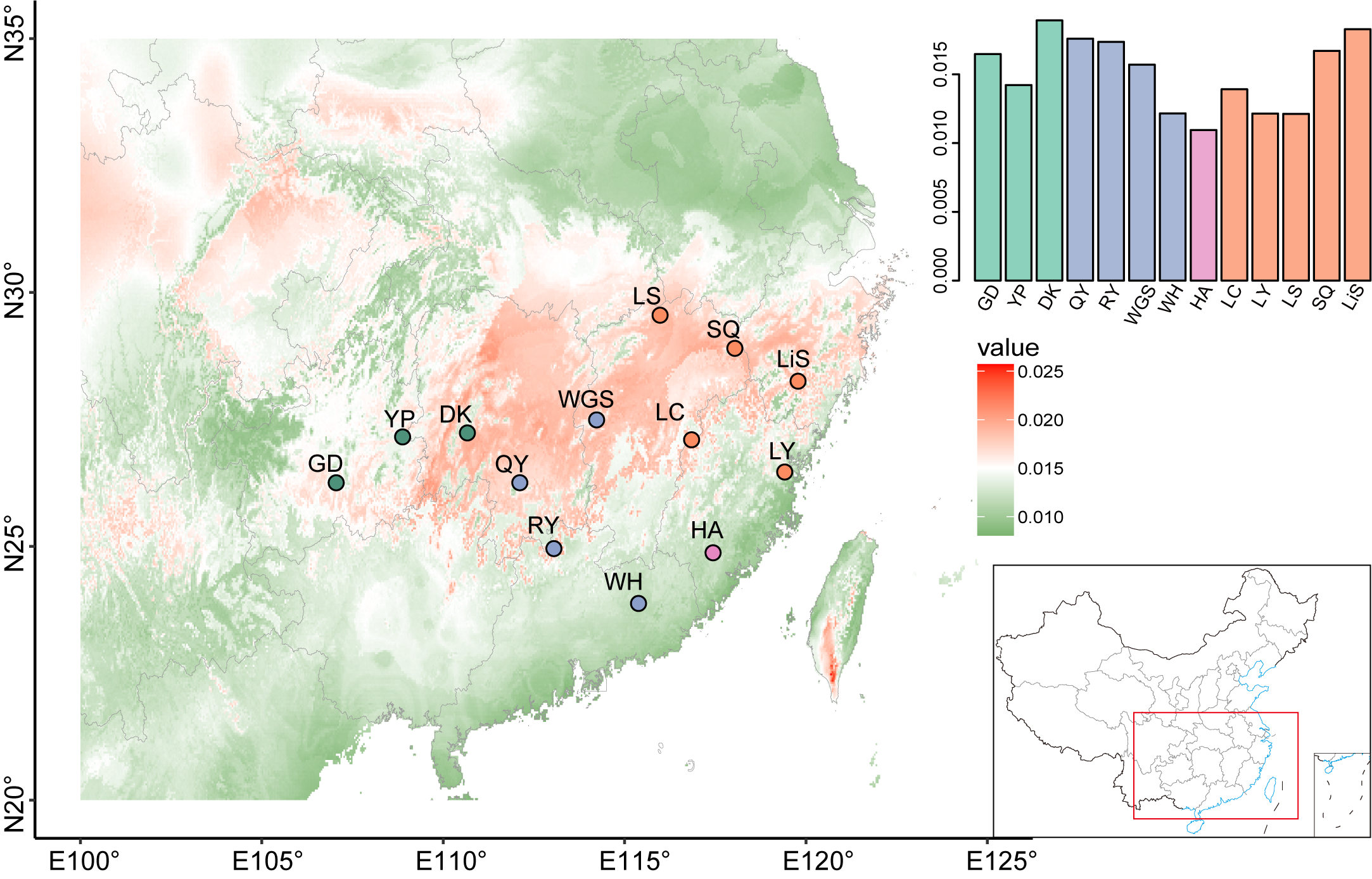

Supplement: Web_Material_uhad031 [file web_material_uhad031.zip › Fig S13_outliers.tif]

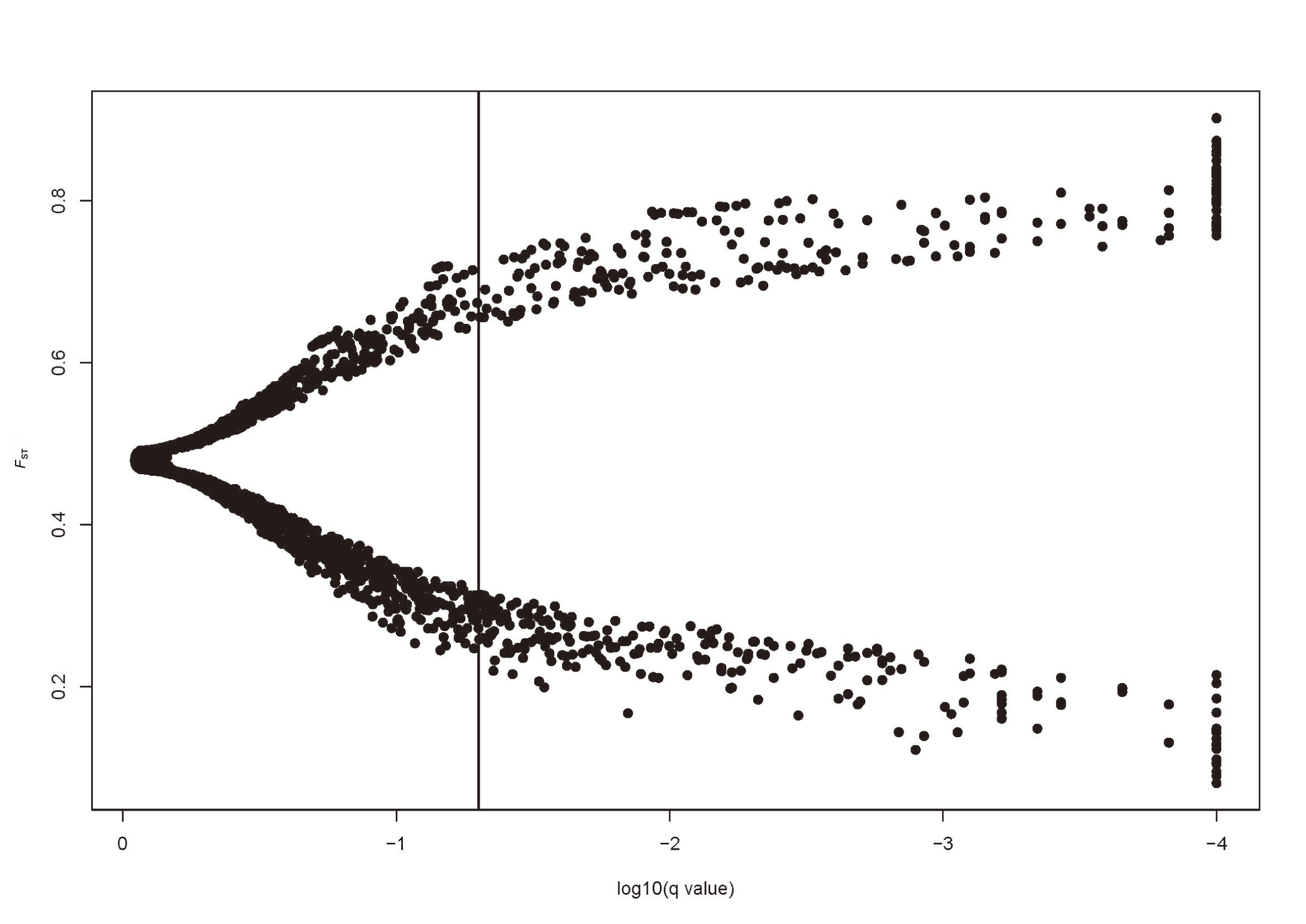

Supplement: Web_Material_uhad031 [file web_material_uhad031.zip › Fig S2.tif]

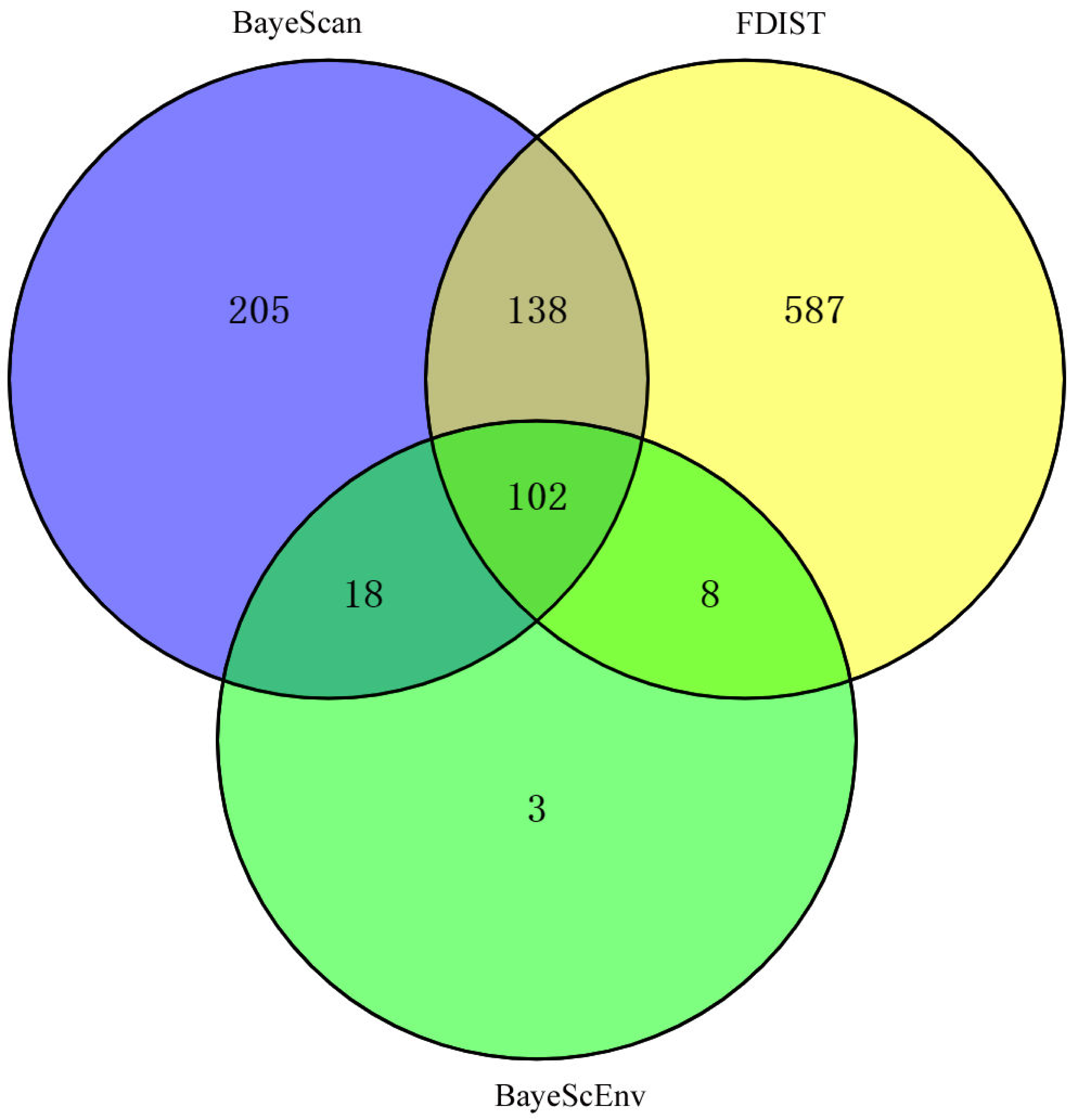

Supplement: Web_Material_uhad031 [file web_material_uhad031.zip › Fig S3.tif]

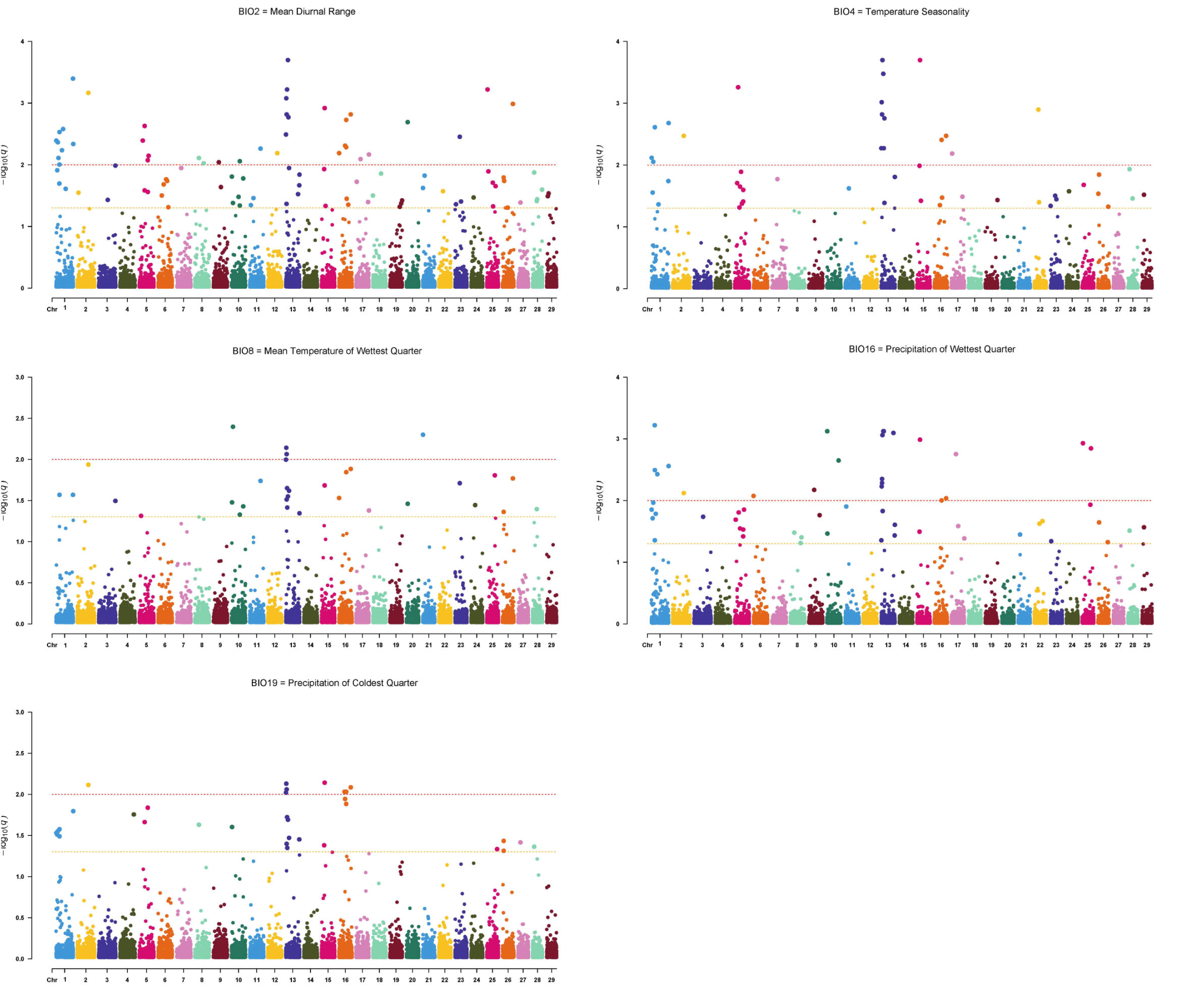

Supplement: Web_Material_uhad031 [file web_material_uhad031.zip › Fig S4.tif]

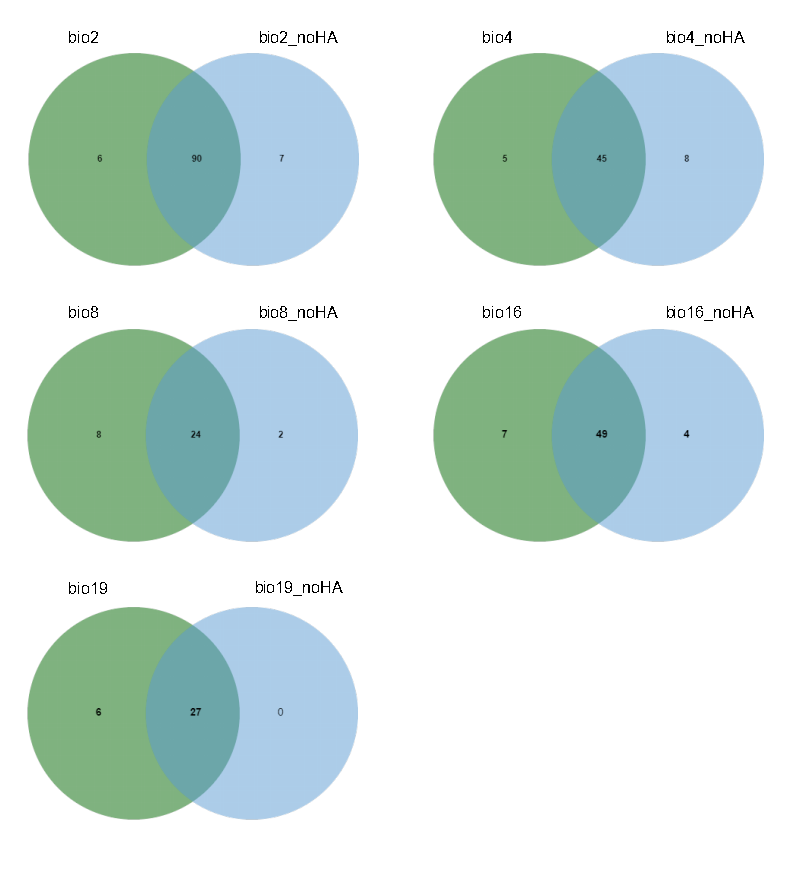

Supplement: Web_Material_uhad031 [file web_material_uhad031.zip › Fig S5 noHAvs.tif]

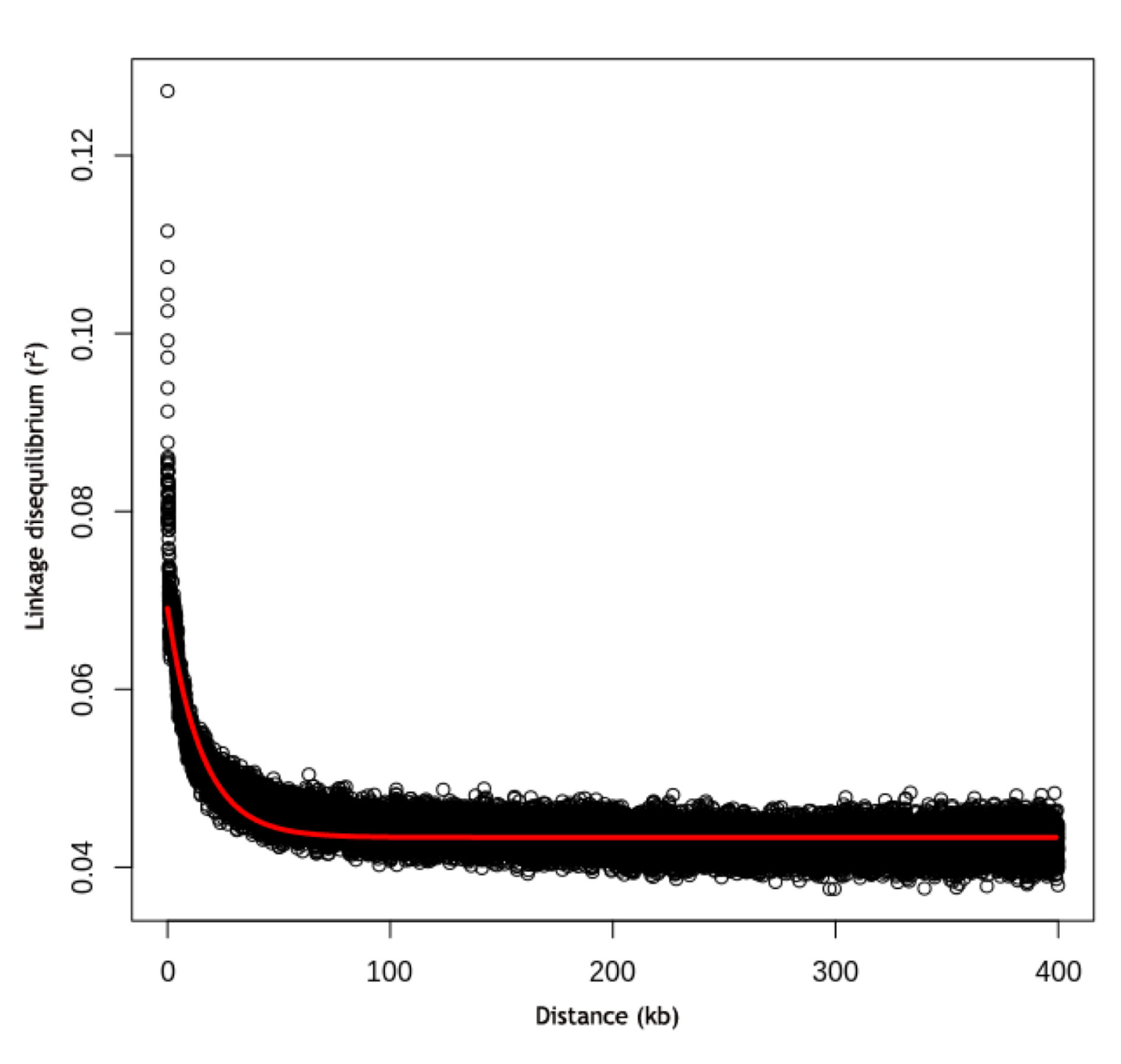

Supplement: Web_Material_uhad031 [file web_material_uhad031.zip › Fig S6_LD.tif]

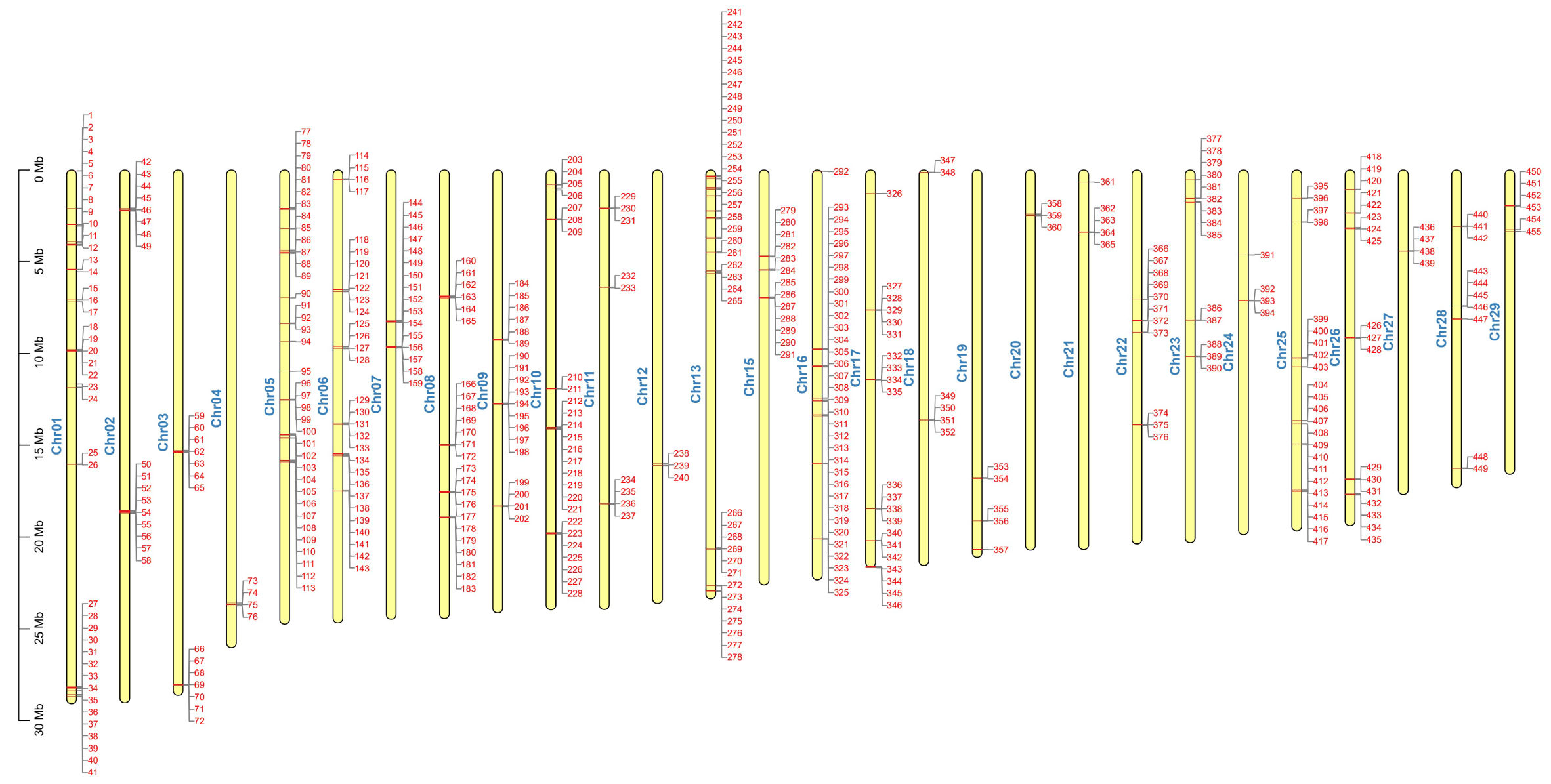

Supplement: Web_Material_uhad031 [file web_material_uhad031.zip › Fig S7_loci.tif]

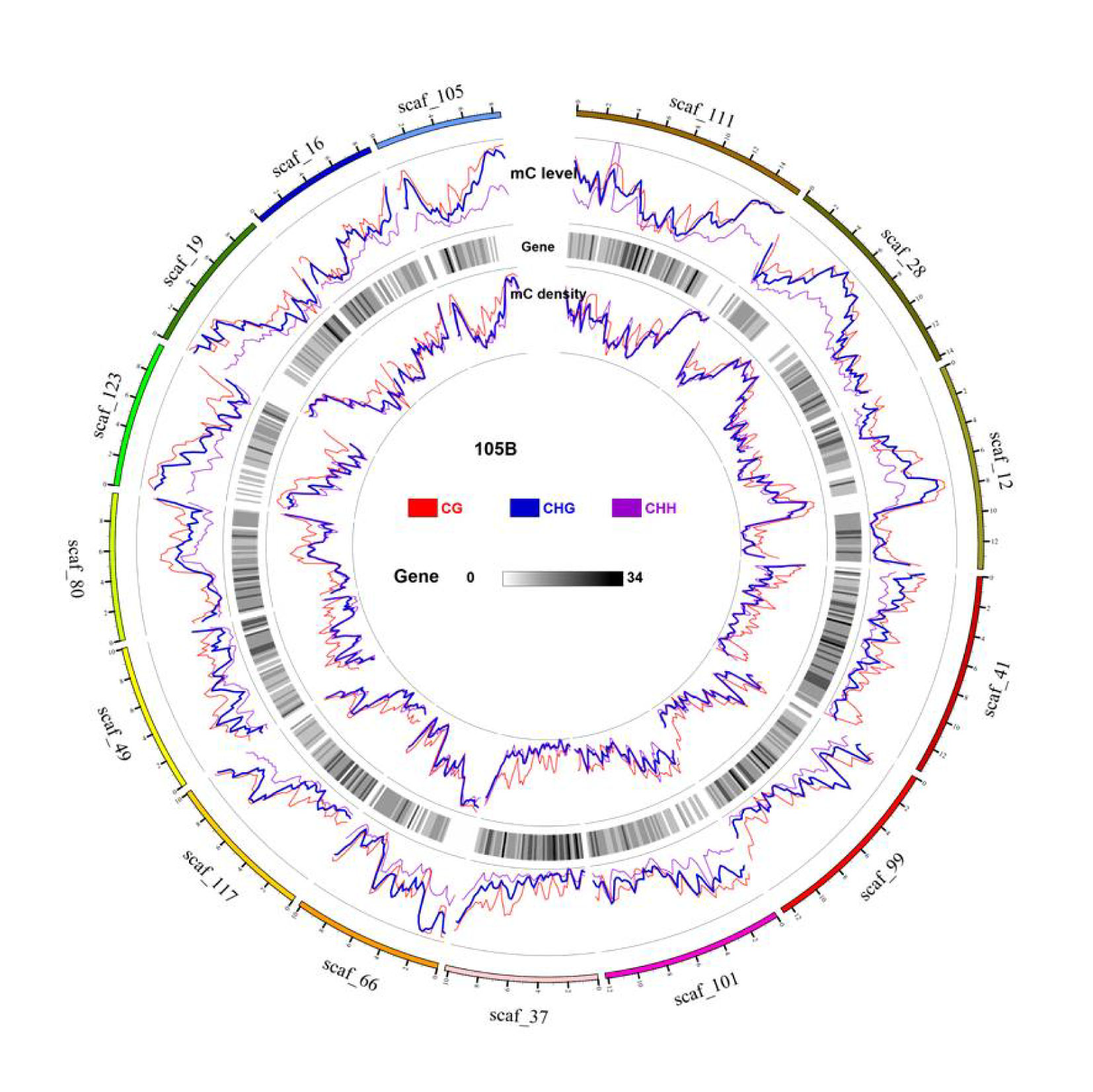

Supplement: Web_Material_uhad031 [file web_material_uhad031.zip › Fig S8_bs.tif]

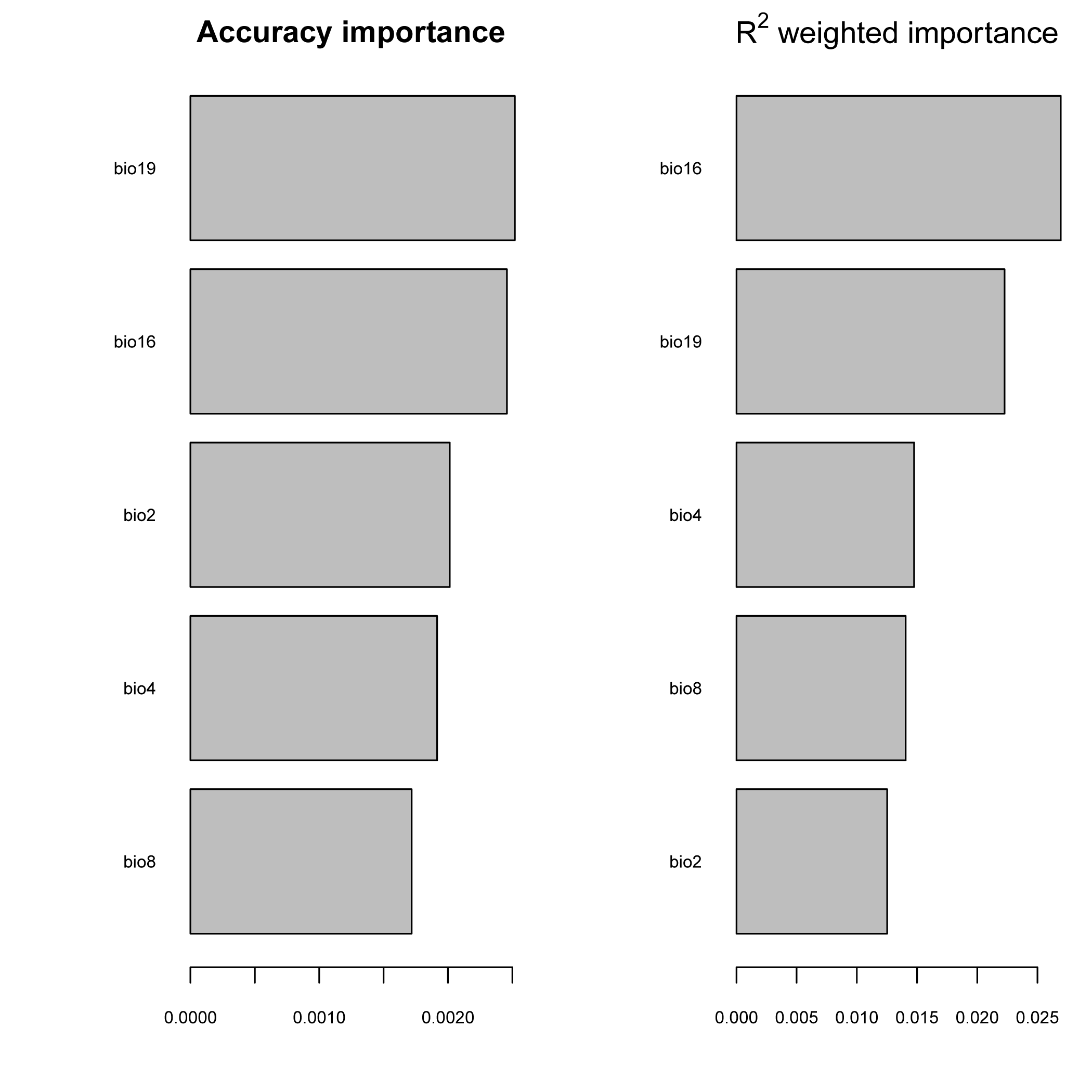

Supplement: Web_Material_uhad031 [file web_material_uhad031.zip › Fig S9_importance.tif]
